# Supplementary material for: A comprehensive evaluation of an artificial intelligence based digital pathology to monitor large-scale deworming programs against soil-transmitted helminths: A study protocol
Source: PLoS One. 2024 Oct 28;19(10):e0309816. doi: 10.1371/journal.pone.0309816 (PMC11515989; doi:10.1371/journal.pone.0309816)
Supplement: S2 File — (PDF) [file pone.0309816.s002.pdf]

Information sheet (English version) for Parents/ guardians

Part I: Instruction: this form is prepared for the parents /guardian of the children in ____________________ (PLEASE INSERT THE NAME OF THE SCHOOL) who will participate in the proposed study.

Investigators:

____ (PLEASE INSERT THE INVESTIGATORS)

Organizations:

____ (PLEASE INSERT YOUR ORGANIZATION)

Sponsor: Enaiblers

Project Title: A comprehensive evaluation of an artificial intelligence based digital pathology to monitor large-scale deworming programs against soil-transmitted helminths

This informed consent has two parts:

Part I: Information sheet

Part II: Certificate of consent

Read and give a copy of the completed informed consent form to the participants.

Part I: Information sheet

Introduction:

Hello! My name is ___________________ (PLEASE INSERT NAME OF PERSON INFORMING the parents/guardians) and I am working with researchers from ______________________ (PLEASE INSERT YOUR ORGANIZATION). We are performing a study to evaluate a new technology to diagnose intestinal worm infections.

Purpose of the research:

Worm infections cause the children to be malnourished, anemic and reduce their growth and development. To combat these worm infections, the World Health Organization recommends large-scale school-based deworming programs. In these programs, school children are periodically (once or twice a year) dewormed. These deworming programs have recently received increased political commitment, resulting in a worldwide increase in drug donations. To verify whether one can scale down these programs, the occurrence of intestinal worms in school children is periodically assessed. The current standard diagnostic is to make a stool smear and to manually count the worm life stages deploying a microscope. However, counting eggs in stool is time consuming and requires laboratories with experienced people that can both perform the surveys and report the results to the health institutions (e.g, Ministry of Health and World Health Organization). The purpose of this study is therefore to validate a new technology that allows automating egg counting in stool smears by Artificial Intelligence (AI), analyzing of data and reporting of the results.

Thus, we are asking children aged between 5-14 from some schools to participate. Your school has been selected. Therefore, we invite you to help us by taking part in this study and hence contribute to making deworm programs worldwide more sustainable.

Procedures:

After agreeing that your child can take part, one or more of our research staff will visit the school on a certain day and give your child a small plastic container and ask him/her to produce a fresh stool sample (at least 5 g). We will make a smear from these samples, in which professionals will look for worm life stages using either a standard microscope or

AI after imaging the smear. If worm life stage are diagnosed in your child using standard microscope, it will will receive an anti-worm drug (single oral pill of _________ (PLEASE INSERT ACTIVE COMPOUND) ______ (PLEASE INSERT DOSE) mg that your child will need to swallow) that will kill any worm that your child may have in his/her intestine. This would improve the general health status, nutritional status and reduce anemia of your child.

Confidentiality:

The information obtained during the study will remain confidential. Disclosure of any of the data people other than those allowed in the Informed Consent form will not be permitted. The results of the research study may be published, but subjects' names or identities will not be revealed. To maintain confidentiality, the investigator will keep records in locked cabinets in a locked room at the office in ________________________________(PLEASE INSERT ORGANISATION) and the results of the tests will be coded to prevent identification of the volunteers. Access to data entered into computerized files will be permitted only for authorized personnel directly involved with the study and will be password protected. Subject-specific information may be provided to responsible local medical personnel only with the subject's permission. Stool collected will not be used for other purposes than validating and developing AI. The remaining stool samples will be safely disposed of.

Risks and Discomfort:

There is little to no risk and discomfort in giving stool sample. Some children may experience a minor side effect after taking the medication. These effects, if they occur, include headache, feeling dizzy, itching and nausea. However, they last for a short time and disappear without intervention themselves. In case of any persistent problem, you are advised to take your child to the nearest Health Centre where staff has been trained and medication is available for this purpose. You can also contact the principal investigator so that you can have access to the physician of the research team.

Benefits:

Children participating in the study will directly benefit by being investigated for worm infections and receiving appropriate treatment. Infected children will improve their general health, nutritional status and reduce anemia. Moreover, the study results will contribute to the development of sustainable deworm programs worldwide.

Incentives:

Every child that has worms will receive an anti-worm drug. However, we will not pay you for taking part in this study. But, we will thank you for your participation.

Right to refuse or withdraw:

You should also know that you are free to withdraw your child from the study at any time and that your choice your choice will not affect the provided education or health services in any way.

Whom to contact:

If you have any questions, you may direct them to the person to whom your child is giving his/her stool sample or whenever you need. Moreover, if you wish to ask questions later, you may contact ______________________ (PLEASE INSERT NAME CONTACT PERSON), in person or by telephone: _____________________ (PLEASE INSERT CONTACT DETAILS).

Part II: Certificate of consent

	I confirm that I have read, or they have read to me the information sheet in a language I understand. I have had the opportunity to consider the information and ask questions and have had these answered satisfactorily.	Yes / No
	I understand that at any time I may withdraw my child from this study without giving reason and without affecting its normal medical care and management.	Yes / No
	I give consent for the data to be stored and shipped overseas.	Yes / No
	I give consent for stool smears and images to be used for future development of Artificial intelligence technologies and my data to be retained and used in future research studies, if approved by an ethics committee.	Yes / No
	I agree that my child takes part in this study.	Yes / No

If illiterate;

Print name of independent literate witness, date and signature of witness (if possible, this person should be selected by the participant and should have no connection to the research team)

Name of witness in print	Signature/Thumbprint	Date (dd/mm/yy)

Name of researcher in print	Signature	Date (dd/mm/yy)

Separate consent form (assent) for children older than 12 years of age participating in the research.

Investigators:

____________________________________ (PLEASE INSERT THE INVESTIGATORS)

Organizations:

____________________________________ (PLEASE INSERT YOUR ORGANIZATION)

Sponsor: Enaiblers

Project Title: A comprehensive evaluation of an artificial intelligence based digital pathology to monitor large-scale deworming programs against soil-transmitted helminths

This informed consent has two parts:

Part I: Information sheet

Part II: Certificate of consent

Read and give a copy of the completed informed assent form to the participants.

Part I: Information sheet

Introduction:

Hello! My name is ____________________ (PLEASE INSERT NAME OF PERSON INFORMING the parents/guardians) and I am working with researchers from _______________________ (PLEASE INSERT YOUR ORGANIZATION). We are performing a study to evaluate a new technology to diagnose intestinal worm infections.

Purpose of the research:

Worm infections cause the children to be malnourished, anemic and reduce their growth and development. To combat these worm infections, the World Health Organization recommends large-scale school-based deworming programs. In these programs, school children are periodically (once or twice a year) dewormed. These deworming programs have recently received increased political commitment, resulting in a worldwide increase in drug donations. To verify whether one can scale down these programs, the occurrence of intestinal worms in school children is periodically assessed. The current standard diagnostic is to make a stool smear and to manually count the worm life stages deploying a microscope. However, counting eggs in stool is time consuming and requires laboratories with experienced people that can both perform the surveys and report the results to the health institutions (eg, Ministry of Health and World Health Organization). The purpose of this study is therefore to validate a new technology that allows automating egg counting in stool smears by Artificial Intelligence (AI), analyzing of data and reporting of the results.

Thus, we are asking children aged between 5-14 from some schools to participate. Your school has been selected. Therefore, we invite you to help us by taking part in this study and hence contribute to making deworm programs worldwide more sustainable.

Procedures:

If you agree to participate, one or more of our research staff will visit the school on a certain day and give you a small plastic container and ask you to produce a fresh stool sample (at least 5 grams). <u>We will make a stool smear from these samples, in which professionals will look for worm life stages using either a standard microscope or AI after imaging the smear. If worm life stage are diagnosed in your stool using standard microscope, </u>you <u>will </u>~~Your child~~ receive an anti-worm drug (single oral pill of _________

(PLEASE INSERT ACTIVE COMPOUND) ______ (PLEASE INSERT DOSE) mg that you swallow) that will kill any worms that you may have in your intestine. This would improve your general health status, nutritional status and reduce anemia. This drug is recommended for the treatment of worm infections.

Confidentiality:

The information obtained during the study will remain confidential. Disclosure of any of the data to people other than those allowed in the Informed Consent form will not be permitted. The results of the research study may be published, but subjects' names or identities will not be revealed. To maintain confidentiality, the investigator will keep records in locked cabinets in a locked room at the office in ______________________________(PLEASE INSERT ORGANISATION) and the results of the tests will be coded to prevent identification of the volunteers. Access to data entered into computerized files will be permitted only for authorized personnel directly involved with the study and will be password protected. Subject-specific information may be provided to responsible local medical personnel only with the subject's permission. Stool collected will not be used for other purposes than validating and developing AI. The remaining stool samples will be safely disposed of.

Risks and Discomfort:

There is little to no risk and discomfort in giving stool sample. Some children may experience a minor side effect after taking the medication. These effects, if they occur, include headache, feeling dizzy, itching and nausea. However, they last for a short time and disappear by without intervention. In case of any persistent problem, you are advised to go the nearest Health Centre where staff has been trained and medication is available for this purpose. You can also contact the principal investigator so that you can have access to the physician of the research team.

Benefits:

Children participating in the study will directly benefit by being investigated for worm infections and receiving appropriate treatment. Infected children will improve their general health, nutritional status and reduce anemia. Moreover, the study results will contribute to the development of sustainable deworm programs worldwide.

Incentives:

Every child will receive an anti-worm drug. However, we will not pay you for taking part in this study. But, we will thank you for your participation.

Right to refuse or withdraw:

You should also know that you are free to withdraw from the study at any time and that your choice will not affect the provided education or health services in any way.

Whom to contact:

If you have any questions, you may direct them to the person to whom you are giving your stool sample. Moreover, if you wish to ask questions later, you may contact ______________________ (PLEASE INSERT NAME CONTACT PERSON), in person or by telephone: _____________________ (PLEASE INSERT CONTACT DETAILS).

Part II: Certificate of consent

	I confirm that I have read, or they have read to me the information sheet in a language I understand. I have had the opportunity to consider the information and ask questions and have had these answered satisfactorily.	Yes / No
	I understand that at any time I may withdraw from this study without giving reason and without affecting its normal medical care and management.	Yes / No
	I give consent for the data to be stored and shipped overseas.	Yes / No
	I give consent for stool smears and images to be used for future development of AI technologies and my data to be retained and used in future research studies, if approved by an ethics committee.	Yes / No
	I agree that to take part in this study.	Yes / No

☐ ☐☐☐

Name of participant in print	Signature/Thumbprint	Date (dd/mm/yy)

Name of researcher in print	Signature	Date (dd/mm/yy)
