# Supplementary material for: A comprehensive evaluation of an artificial intelligence based digital pathology to monitor large-scale deworming programs against soil-transmitted helminths: A study protocol
Source: PLoS One. 2024 Oct 28;19(10):e0309816. doi: 10.1371/journal.pone.0309816 (PMC11515989; doi:10.1371/journal.pone.0309816)
Supplement: S3 File — (PDF) [file pone.0309816.s003.pdf]

A comprehensive evaluation of an artificial intelligence based digital pathology to monitor large-scale deworming programs against soil-transmitted helminths: a study protocol

SUPPLEMENTARY INFO S3. The methodology to determine the required sample sizes to test the project hypotheses around diagnostic performance, repeatability, and reproducibility.

Peter Ward[1,2,3¶], Sara Roose[1¶], Mio Ayana[4], Lindsay A Broadfield[2], Peter Dahlberg[2], Narcis Kabatereine[5], Adama Kazienga[1], Zeleke Mekonnen[4], Betty Nabatte[5], Lieven Stuyver[6], Fiona Vande Velde[1], Sofie Van Hoecke[3], Bruno Levecke[1]*

[1]Department of Translational Physiology, Infectiology and Public Health, Ghent University, Merelbeke, Belgium

[2]Enaiblers AB, Uppsala, Sweden.

[3]IDLab, Department of Electronics and information systems, Ghent University – Imec, Zwijnaarde, Belgium

[4]Institute of Health, Jimma University, Jimma, Ethiopia

[5]Vector Borne and Neglected Tropical Diseases Division, Kampala, Uganda
Department of Translational Physiology, Infectiology and Public Health, Ghent University,
Merelbeke, Belgium

[6]Scientific Advisor

¶ These authors contributed equally to this work.

* Corresponding author. Email: Bruno.levecke@UGent.be (BL)

Introduction

This Supplementary Info describes the applied methodology to calculate the required sample sizes for each of the 8 (***H1.1.-4; H2.H2.4***) hypothesis mentioned in the main document. Generally, we opted to conduct a series of simulation studies over the standard sample size methodologies, as this approach allowed us (i) to better capture the variation in test results that are otherwise difficult to account for (e.g. clinical sensitivity of Kato Katz thick smear increases as a function of eggs in a slide) and (ii) to ensure that both the sample size calculation and the final interpretation of the field data are based on the same statistical approach (e.g. the relative position of confidence intervals (CI) to predefined set of values; see also **Fig 1**). In brief, each of these simulation studies consists of a series of in-silico experiments that are iterated under different conditions (e.g. different sample sizes). Based on this iterative process, we determined the lowest sample size that allowed for confirming the hypothesis in at least 80% of the iterations (= power). The annotated R-script that was used to determine the sample size can be found at the end of this document. To fully align with the main document, we have also structured this Supplementary Info on an experiment basis.

Diagnostic performance

This experiment addresses the following four non-inferiority hypotheses

(1) the clinical sensitivity of KK2.0 to detect low intensity infections is non-inferior to that of KK1.0 for *Ascaris*, *Trichuris* and hookworms (***H1.1***)
(2) the clinical sensitivity of KK2.0 to detect MHI infections is non-inferior to that of KK1.0 for *Ascaris*, *Trichuris* and hookworms (***H1.2***)
(3) the clinical specificity of KK2.0 to detect any intensity infections is non-inferior to that of KK1.0 for *Ascaris*, *Trichuris* and hookworms (***H1.3***)
(4) the clinical specificity of KK2.0 to detect MHI infections is non-inferior to that of KK1.0 for *Ascaris*, *Trichuris* and hookworms (***H1.4***)

For each hypothesis, we assumed an equivalence level of 5-point percent. In other words, the lower limit of the CI around the difference (KK2.0 – KK1.0) should be at least -5% (**Fig 1**).

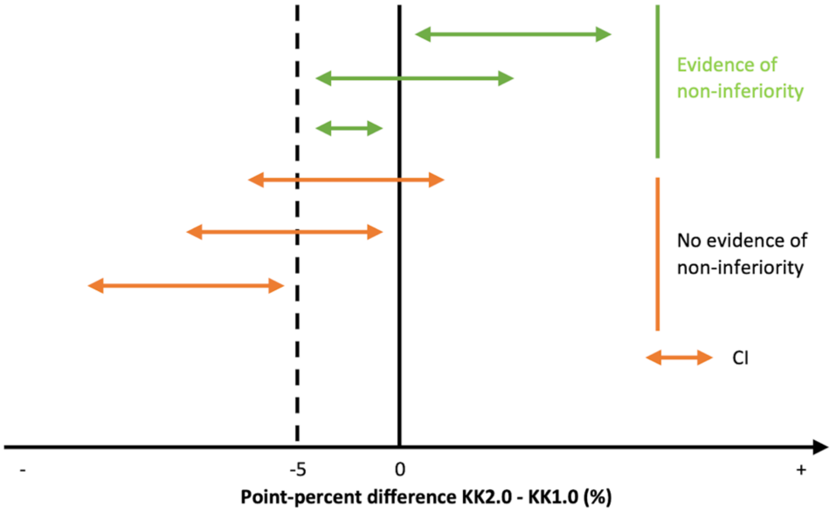

Fig 1. Overview of the different outcome scenarios based on a random sample and its corresponding CI. This figure illustrates the different outcome scenarios around the difference in performance between KK2.0 and KK1.0 based on the CI when testing the hypothesis that the performance of KK2.0 is non-inferior to that of KK1.0.

Clinical sensitivity to detect low infection intensities

Data generation methodology

In this first simulation study, we accounted for (i) a varying clinical sensitivity as a function of the number of eggs in a smear; (ii) a correlation between test results of KK1.0 and KK2.0 on the same smear, (iii) and helminth specific fecal egg count (FEC; expressed as eggs per gram of stool (EPG)) thresholds defining low intensity infections. It is well-known that the clinical sensitivity increases when more eggs are present in a slide [1]. We therefore introduced for each test x the probability $\pi_{i,x}^{+}$ of detecting at least one egg (resulting in positive test result) in smear i in our data generate process. The value for this parameter is a function of the probability $p_{test,x}$ of a single egg being detected when presented to a test x (KK1.0: human eye; KK2.0: AI + human eye) and a number n_i eggs in a smear i (**Eq 1**).

$$\pi_{i,x}^{+} = 1 - \ (1 - p_x)^{n_i} \text{ (Eq 1)}$$

Fig 2 further illustrates the change $\pi_{i,x}^{+}$ over different values of n_i (1 – 10 eggs) for six fictive tests (1-5), each with a different p_x ($p_1 = 0.5$, $p_2 = 0.6$, $p_3 = 0.7$, $p_4 = 0.8$, $p_5 = 0.9$, $p_6 = 0.95$). Generally, the figure confirms that our data generation process accounts for a varying clinical sensitivity as a function of the number of eggs in a smear, and that, even in the worst-case scenario ($p_1 = 0.5$), $\pi_{i,x}^{+}$ equals 1 when 10 eggs are in a slide.

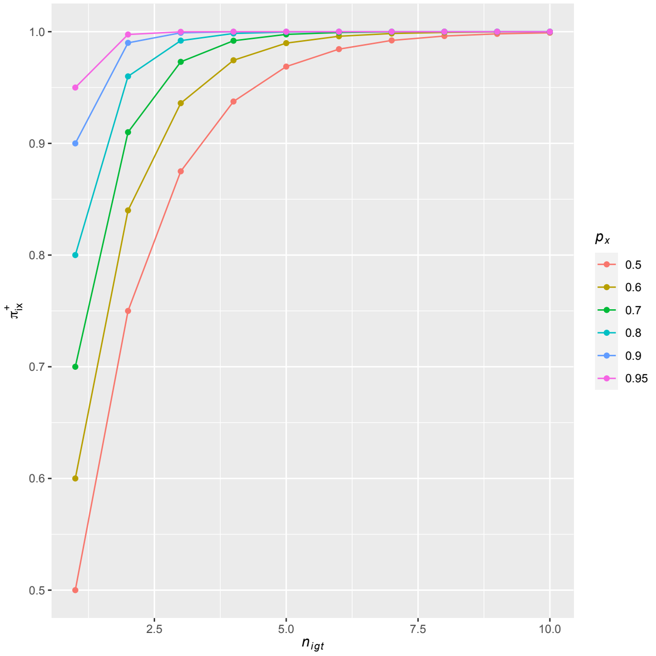

Fig 2. The change $\pi^+_{i,x}$ over different values of n_i eggs. This plot illustrates the change in the probability of having a positive test result ($\pi^+_{i,x}$) over different values of n_i (1 – 10 eggs) for six fictive tests 1 – 6, each with a different p_x ($p_1 = 0.5$, $p_2 = 0.6$, $p_3 = 0.7$, $p_4 = 0.8$, $p_5 = 0.9$, $p_6 = 0.95$).

Given that we will process the same KK smear by two tests (KK1.0 *vs.* KK2.0), test results will not be independent. In other words, we are dealing with paired data. We therefore assumed the same p_x for both KK1.0 and KK2.0 and set n_i eggs in a smear i equal to the ground truth ($n_{i,gt}$) in the data generating process. In **Table 1**, we provide a toy example of fictive test results for KK1.0 and KK2.0 for N_{tot} slides where we assumed $p_{test,x} = 0.9$ and that results ($t^+_{i,x}$, positive *vs.* negative) for a test x on smear i follows a Bernoulli trial with a probability of a positive test result equal to $\pi^+_{i,x}$ (**Eq 2**).

$$t^+_{i,x} \sim Bernoulli\left(\pi^+_{i,x}\right) \textbf{ (Eq 2)}$$

In this fictive toy example, there was an agreement in nine out of the 10 slides. In one slide, KK2.0 resulted in a positive test result, while KK1.0 provided a negative test result. Given the ground truth is non-zero, the clinical sensitivity in this example is 70.0% for KK1.0 and 80.0% for KK2.0. To make any inference on the clinical sensitivity of KK2.0 compared to KK1.0, we calculated the corresponding 95%CI around the difference in sensitivity (KK2.0 – KK1.0). For this, we applied the formulae described by Newcombe for paired data [2]. In the toy example, the 95%CI around the 10-point percent difference was (-10.0; 31.0), and hence we cannot make any conclusions on the inferiority (see *inconclusive* in **Fig 1**).

Table 1. A toy example of the data generation process. This table represents a toy example of the data generation process to determine the required sample size to test the hypothesis that clinical sensitivity of KK2.0 is non-inferior to that of KK1.0.

ID slide	$n_{i,gt}$	KK1.0	KK2.0
1	5	1	1
2	2	0	1
3	10	1	1
4	9	1	1
5	5	1	1
6	1	0	0
7	3	1	1
8	1	0	0
9	2	1	1
10	2	1	1
Sensitivity		70.0%	80.0%
Difference (95%CI)		10.0% (-10.0; 31.0)	

To classify the intensity of infections, we applied the FEC thresholds by WHO for each of the different helminth species. As illustrated by **Table 2**, these thresholds are helminth specific [3]. Given this difference in infection intensity thresholds and the variation in clinical sensitivity as a function of $n_{i,gt}$, we determined the required sample size for each helminth species separately. To obtain helminth specific $n_{i,gt}$ for N_{tot} slides, we drew a random sample (with replacement) of N from any value between 1 and the highest possible FEC that represents a low intensity infection divided by 24 (= $n_{max,low}$). This division is required because the FEC thresholds are expressed in EPG, while our data generation process is based on the raw counts in smear of 1/24 gram of stool. To ensure integer values of $n_{i,gt}$ we rounded $n_{max,low}$ to the nearest lowest integer value.

Table 2. The FEC thresholds defining low, moderate and heavy STH infections. This table summarizes the WHO FEC (in EPG) thresholds to classify the intensity of STH infections into low, moderate, and high. $n_{i,gt}$, represents the corresponding raw egg (= lowest integer of FEC / 24 EPG) in a single smear i.

Helminth	Low		Moderate		High
	FEC (EPG)	$n_{max,low}$ (eggs)	FEC (EPG)	$n_{max,moderate}$ (eggs)	FEC (EPG)
Ascaris	1 – 4,999	208	5,000 – 49,999	2,083	≥50,000
Trichuris	1 – 999	41	1,000 – 9,999	416	≥10,000
Hookworm	1 – 1,999	83	2,000 – 3,999	166	≥4,000

In our final simulation study, explored a total of 819 scenarios, reflecting different combinations of $n_{max,low}$ (*Ascaris*: 208 eggs; *Trichuris*: 41; hookworm: 83), $p_{test,x}$ (0.5, 0.6, 0.7, 0.8, 0.9, 0.95 and 1) and N_{tot} (from 60 to 250 with steps of 5 smears). Since little is known on p_x for both KK1.0 and KK2.0, and given the impact on the required sample size, we explored different values for $p_{test,x}$. For any value of p_x we assumed that this was the same for both KK1.0 and KK2.0. The power (proportion of the iterations in which it was concluded that the sensitivity of KK2.0 was non-inferior based on the CI) for each combination was determined based on 500 iterations. Given the multiple hypothesis testing (one for each STH, three in total), and a single sided hypothesis testing, the level of significance was set at 0.03 (= 0.1/3).

Required sample sizes

Fig 3 provides an overview of the power as a function of the number of slides for the different values of $p_{test,x}$ for each of the three STH species separately. Overall, the power increased as a function of N_{tot} and p_x across each STHs. For the same power, N_{tot} was lowest for *Ascaris* and highest for *Trichuris*. Indeed, for a power of ≥80% and when p_x was set at 0.9, the required sample size was 105 for *Ascaris*, 125 for hookworms and 160 for *Trichuris*.

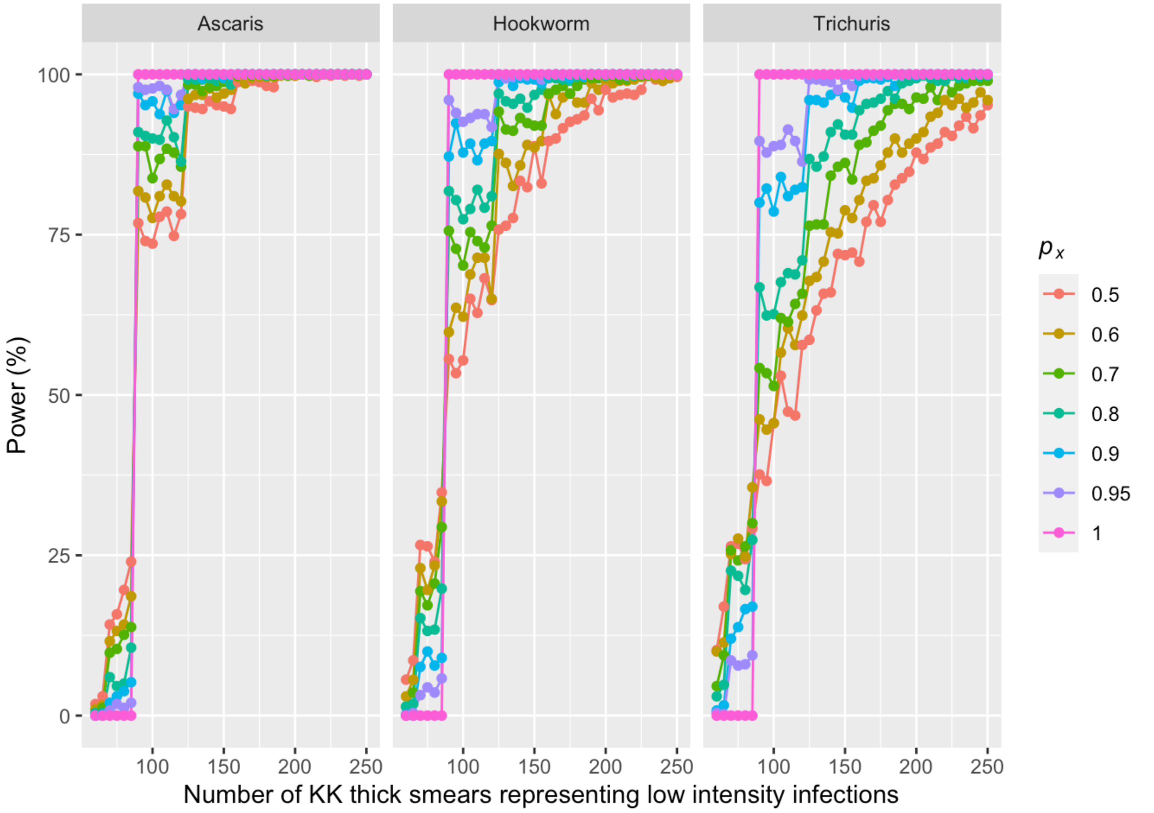

Fig 3. The power to test the hypothesis that clinical sensitivity of KK2.0 to detect low intensity infections is non-inferior to that of KK1.0. This plot illustrates the power as a function of increasing smears for seven values of p_x and three STHs.

Table 3 further summarizes the lowest possible sample size that results in a power of at least ≥80% across the seven values p_x and the three STHs.

Table 3. The required number of smears representing low intensity infections to draw reliable conclusions on the non-inferiority sensitivity of KK2.0 to detect low intensity *Ascaris*, *Trichuris* and hookworm infections.

p_x	*Ascaris*	*Trichuris*	Hookworm
0.50	125	180	140
0.60	125	180	140
0.70	125	180	140
0.80	105	160	125
0.90	105	160	125
0.95	105	160	125
1.00	90	140	125

Today little is known about p_x for both KK1.0 and KK2.0. Unpublished *Ascaris* spiking experiments, indicated that the detection limit of KK1.0 is 5 eggs (95% of the slides with 5

eggs will result in a positive test result), which corresponds with a p_x equal to 0.5 (see also **Fig 2,** where $\pi_{i,x}^{+}$ exceeds 0.95 for a p_x equal to 0.5 when n_i equals 5). Based on our findings (the current simulation study and the unpublished spiking experiment), we recommend to include at least 125 slides *Ascaris*, 180 for *Trichuris* and 140 for hookworms.

Clinical sensitivity to detect moderate-to-heavy intensity infections

Data generation methodology

In this second simulation study, we had to amend the aforementioned data generation process in three ways. First, we had to generate egg counts for each test ($n_{i,x}$), separately. To this end, we assumed that $n_{i,x}$ follows a binomial distribution with $n_{i,gt}$ Bernoulli trials and a probability of success equal to p_x (**Eq 3**).

$$n_{i,x} \sim Binom\left(n_{i,gt}, p_x\right) \quad \textbf{(Eq 3)}$$

Second, we had to determine the test result $t_{i,x}^{MHI+}$ based on how $n_{i,x}$ relates to the threshold of moderate-to-heavy intensity (MHI) infections ($n_{max,low} + 1$) (**Eq 4**), where $t_{i,x}^{MHI+} = 1$ reflects a truly positive test result for an MHI infection and $t_{i,x}^{MHI+} = 0$ represents a false negative test result.

$$t_{i,x}^{MHI+} \begin{cases} 1 \; if \; n_{i,x} \geq n_{max,low} + 1 \\ 0 \; if \; n_{i,x} < n_{max,low} + 1 \end{cases} \quad \textbf{(Eq 4)}$$

Third, we had to adapt the range of $n_{i,gt}$, reflecting the expected range of MHI infections. Indeed, While $n_{i,gt}$ could not exceed $n_{max,low}$ in the first simulation study, $n_{i,gt}$ could now take any integer value between $n_{max,low}$ + 1 and the highest possible FEC that represents a moderate intensity infection divided by 24 (= $n_{max,moderate}$). Although we could have extended this range to the highest possible FEC that represents a heavy intensity infection divided by 24 (= $n_{max,heavy}$), we opted not to do so. This is because (i) we already cover a wide range of possible $n_{i,gt}$ between $n_{max,low} + 1$ and $n_{max,moderate}$, (ii) we expect both the most negative test results when $n_{i,gt}$ approaches $n_{max,low}$, and (iii) the moderate infection intensity infections will cover most of the moderate-to-heavy intensity infection cases.

To further illustrate these changes, we have worked out a toy example in **Table 4**. This table provides the fictive test results ($n_{i,x}, t_{i,x}^{MHI+}$) for KK1.0 and KK2.0 for 10 slides where we assumed $p_x = 0.5$, and $n_{i,gt}$ varying between 10 ($= n_{max,low} + 1$) and 100 ($= n_{max,moderate}$) (**Eq 2**).

Table 4. A toy example of the data generation process. This table represents a toy example of the data generation process to determine the required sample size to test the hypothesis that KK2.0 is non-inferior to KK1.0 to detect MHI infections

**ID slide**	$n_{i,gt}$	**KK1.0**		**KK2.0**	
		Egg counts	MHI	Egg counts	MHI
1	45	22	1	22	1
2	67	29	1	34	1
3	88	46	1	45	1
4	88	49	1	35	1
5	29	11	1	12	1

6	97	43	1	44	1
7	15	8	0	11	1
8	30	19	1	20	1
9	73	26	1	33	1
10	54	27	1	28	1
		Sensitivity	90.0%		100%
		Difference (95%CI)			10.0% (-19.0; 40.0)

In our final simulation study, explored a total of 918 scenarios, reflecting different combinations of $n_{max,low}$ (*Ascaris*: 208 eggs; *Trichuris*: 41; hookworm: 83), $n_{max,moderate}$ (*Ascaris*: 2,038 eggs; *Trichuris*: 416; hookworm: 166), p_x (0.5, 0.6, 0.7, 0.8, 0.9, 0.95) and N_{tot} (from 100 to 350 with steps of 5 smears). Since little is known on $p_{test,x}$ for both KK1.0 and KK2.0, and given the impact on the required sample size, we explored different values for p_x. For any value of p_x, we assumed that this was the same for both KK1.0 and KK2.0. The power (proportion of the iterations in which it was concluded that the sensitivity of KK2.0 was non-inferior based on the CI) for each combination was determined based on 500 iterations. Given the multiple hypothesis testing (one for each STH, three in total) and a single sided hypothesis testing, the level of significance was set at 0.03.

Required sample sizes

Fig 4 provides an overview of the power as a function of the number of smears for the different values of p_x for each of the three STH species separately. Overall, the power increased as a function of N_{tot} and p_x across each STH. For the same power, N_{tot} was lowest for *Ascaris* and highest for hookworm. Indeed, for a power of ≥80% and when p_x was set at 0.9, the required sample size was 100 for *Ascaris*, 125 for *Trichuris* and 195 for hookworms.

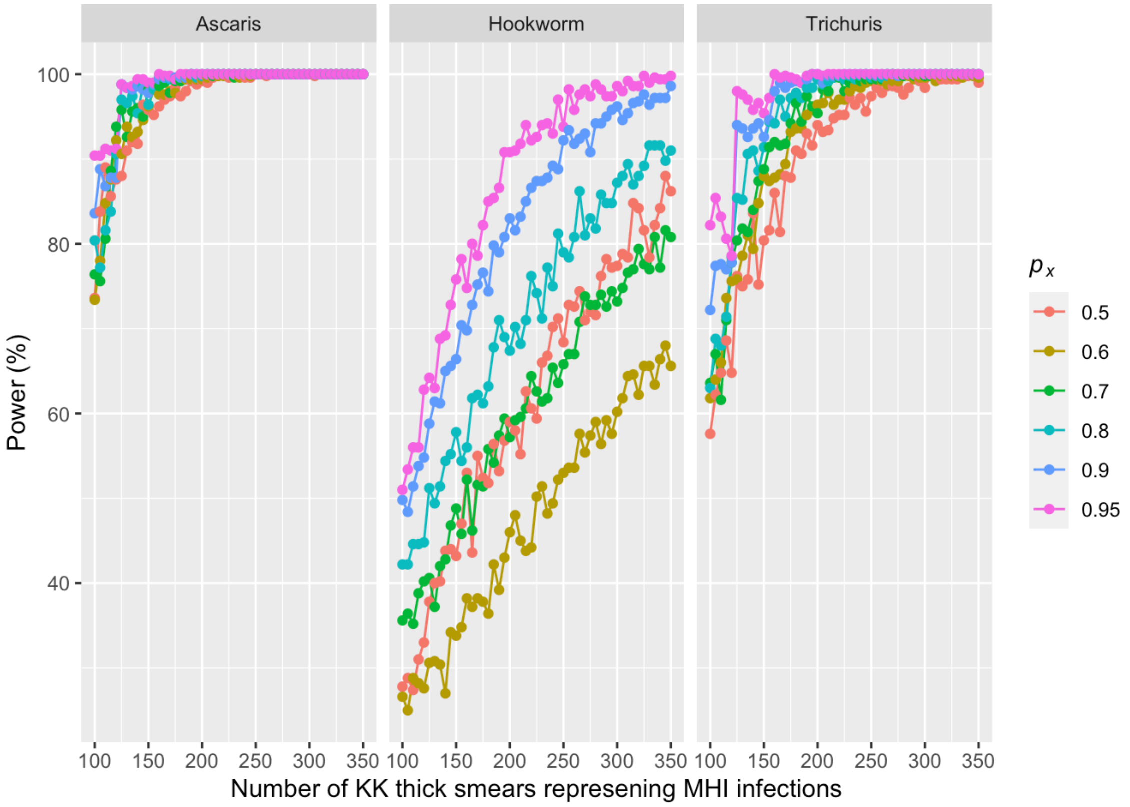

Fig 4. The power to test the hypothesis that clinical sensitivity of KK2.0 to detect low MHI infections is non-inferior to that of KK1.0. This plot illustrates the power as a function of increasing smears for six values of p_x and three STHs.

Table 5 further summarizes the lowest possible sample size that results in a power of at least ≥80% across the seven values p_x and the three STHs.

Table 5. The required number of smears to draw reliable conclusions on the non-inferiority sensitivity of KK2.0 to detect MHI *Ascaris*, *Trichuris* and hookworm infections.

p_x	*Ascaris*	*Trichuris*	Hookworm
0.50	105	150	>350
0.60	110	145	>350
0.70	110	125	>350
0.80	110	125	260
0.90	100	125	195
0.95	100	125	175

Today, little is known about p_x for both KK1.0 and KK2.0. Unpublished *Ascaris* spiking experiments, indicated that the egg counts based on KK1.0 were 0.60 to 0.70 times the number of spiked eggs, suggesting a $p_{test,x}$ equal to 0.6 or 0.7. Based on our findings (the current simulation study and the unpublished spiking experiment), we recommend including at least 110 slides *Ascaris*, 145 for *Trichuris* and >350 for hookworms each.

Clinical specificity to detect any and moderate-to-high intensity infections

Given that we (i) applied the same data generation process that (ii) is also generic for the different helminth species, we will combine the sample size calculation for the different hypotheses linked to clinical specificity in this section.

Data generation methodology

In contrast to the methodology for the hypothesis concerning clinical sensitivity for which individual test results ($t_{i,x}^{+}$, $t_{i,x}^{MHI+}$) could be written as a function of $n_{i,gt}$, this revealed to be more challenging for the specificity. We therefore opted to generate the different combination test results between two tests instead. Generally, there are four test result combinations when deploying two tests on the same truly negative slides; both tests are (i) negative or (ii) positive, or (iii) Test 1 is shows a positive test result, while the Test 2 a negative test result and (iv) vice versa. In **Table 6A**, we assigned a probability to each of these test result combinations (p_{++},p_{--},p_{+-}, and p_{-+}) so that the sum of these probabilities equals 1. From this 2x2 table, we can also deduce the specificity of both Test 1 ($sp_1 = p_{-+} + p_{--}$) and Test 2 ($sp_2 = p_{+-} + p_{--}$). To generate test results across 2 tests, we can now draw a random sample of N_{tot} from the multinomial distribution with four groups each with the corresponding probabilities p_{++},p_{--},p_{+-}, and p_{-+}. An example, for a random sample of 10 negative slides form a multinomial distribution for which $p_{++} = p_{--} = p_{+-} = p_{-+} = 0.25$ is illustrated in **Table 6C**.

Table 6. The different test result combinations when two tests are deployed on the same set of negative slides

A

	Test 2		
Test 1	+	-	
+	p_{++}	p_{+-}	$1 - sp_1$
-	p_{-+}	p_{--}	sp_1
	$1 - sp_2$	sp_2	1

B

	Test 2		
Test 1	+	-	
+	N_{++}	N_{+-}	N_{1+}
-	N_{-+}	N_{--}	N_{1-}
	N_{2+}	N_{2-}	N_{tot}

C

	Test 2		
Test 1	+	-	
+	3	1	4
-	4	2	6
	7	3	10

This table can now also be translated into a dataset, similarly as that illustrated in **Tables 1** and **4**, and hence we can apply the aforementioned approach to both the determine the power (proportion of the 500 iterations in which it was concluded that the specificity of KK2.0 was non-inferior based on the CI). Although one would need to provide values for four different parameters (p_{++},p_{--},p_{+-}, and p_{-+}), we made two assumptions that allowed us to reduce the input parameters to one, namely (i) both tests have an equal specificity sp and (ii) p_{++},p_{--},p_{+-}, and p_{-+} are defined as a function of sp. For the latter, we even defined three different scenarios, each representing a different concordance in false positive test results across both tests (perfect, moderate and no concordance; **Table 7**). For the perfect concordance, both p_{+-}, and p_{-+} were set at zero, while p_{++} equalled $1 - sp$. For a moderate concordance, p_{+-}, p_{-+} , p_{++} were set at $(1 - sp)/3$. When there was no concordance in test results, p_{++} was set at zero, while both p_{+-}, and p_{-+} equalled $(1 - sp)/2$. In **Table 7**, we further illustrate these levels of concordance when sp was set at 0.95. In the final simulation study, we varied sp from 0.90 to 1 with a 0.01 interval and N_{tot} from 100 to 350 with steps of 5 smears, resulting in 1,683 combinations.

Table 7. The different scenarios of concordance in false positive test results between Test 1 and Test 2.

No concordance

	Test 2		
Test 1	+	-	
+	0	$\frac{1-sp}{2}$	$\frac{1-sp}{2}$
-	$\frac{1-sp}{2}$	sp	$\frac{1-sp}{2} + sp$
	$\frac{1-sp}{2}$	$\frac{1-sp}{2} + sp$	1

Moderate concordance

	Test 2		
Test 1	+	-	
+	$\frac{1-sp}{3}$	$\frac{1-sp}{3}$	$2\left(\frac{1-sp}{3}\right)$
-	$\frac{1-sp}{3}$	sp	$\frac{1-sp}{3} + sp$
	$2\left(\frac{1-sp}{3}\right)$	$\frac{1-sp}{3} + sp$	1

Perfect concordance

	Test 2		
Test 1	+	-	
+	$1-sp$	0	$1-sp$
-	0	sp	sp
	$1-sp$	sp	1

	Test 2		
Test 1	+	-	
+	0	0.025	0.025
-	0.025	0.950	0.975
	0.025	0.975	1

	Test 2		
Test 1	+	-	
+	0.050/3	0.05/3	1.00/3
-	0.050/3	0.950	0.950 + 0.05/3
	1.000/3	0.950 + 0.05/3	1

	Test 2		
Test 1	+	-	
+	0.05	0	0.05
-	0	0.95	0.95
	0.05	0.95	1

Required sample sizes

Fig 5 provides an overview of the power as a function of the number of smears for the different levels of concordance. Overall, the power for a given sample size increased as a function of increased concordance in false positive test results and specificity.

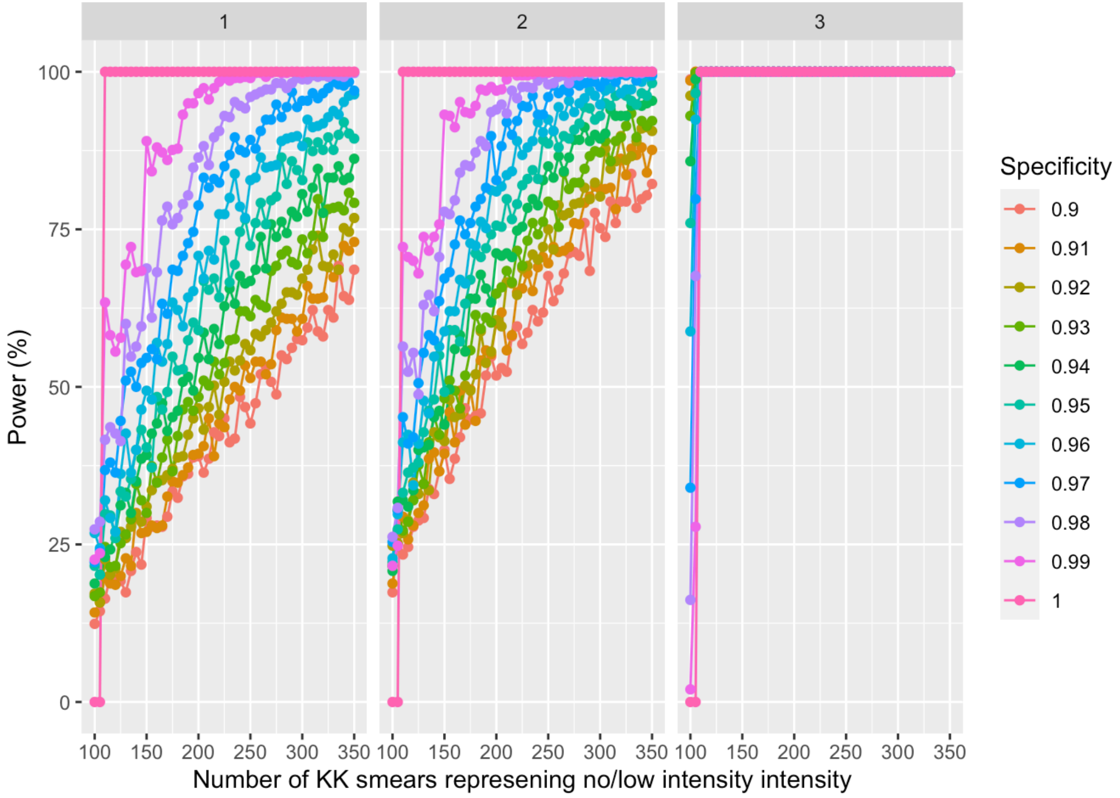

Fig 5. The power to test the hypothesis that clinical specificity of KK2.0 to detect any or MHI infections is non-inferior to that of KK1.0. This plot illustrates the power as a function of increasing number of smears for 11 values specificity and three levels of concordance in false positive test results (1: no concordance; 2: moderate concordance; 3: perfect concordance).

Table 8 further summarizes the lowest possible sample size that results in a power of at least ≥80% across the seven values $p_{test,x}$ and the three levels of concordance in false positive test results. For testing the hypothesis around the specificity of any intensity infections, we assumed a specificity of at least 95% [4] and a moderate concordance in false positive test results, resulting in a sample size of least 225 slides without any eggs for a given STH. For testing the hypothesis around the specificity of MHI infections, we assumed a specificity of at least 98% and a moderate concordance in false positive test results, resulting in a sample size of least 165 slides representing low intensity infections for a given STH.

Table 8. The required number of smears to draw reliable conclusions on the non-inferiority specificity of KK2.0 to detect *Ascaris*, *Trichuris* and hookworm infections of any intensity and MHI.

Specificity	No concordance In false positive test results	Moderate concordance In false positive test results	Perfect concordance In false positive test results
0.90	>350	>350	100
0.91	>350	290	100
0.92	>350	295	100
0.93	>350	265	100
0.94	325	245	100
0.95	275	225	105
0.96	250	195	105

0.97	205	190	110
0.98	190	165	110
0.99	150	150	110
1.00	110	100	100

Repeatability and producibility

Data generation methodology

To verify whether the repeatability and reproducibility is at least 99% for the scanner set-up (***H2.1***), the AI verification process (***H2.2***), the complete KK2.0 is at least 99% (***H2.3***), we conducted a simulation study where we determined the number of KK thick smears that resulted in a lower limit of the Wald CI that is at least 95% in 80% (= power) of the iterations when the true underlying probability of success equals 99%. To determine this, we performed a simulation study in which we first generated 500 x N_{tot} Bernoulli trials, where the probability for success was set at 0.99. Subsequently, we determined for each set of *N* Bernoulli trials the CI Wald's (asymptotic) confidence interval and verified whether the lower limit of the confidence interval was at least 90%. Finally, we repeated this process for *N* varying between 60 and 250 (with increments of 5 smears). Given that we are testing both repeatability and reproducibility at same time for each process, and that we are testing for non-inferiority, we set the level of significance at 0.1/2. In addition, we explored the power when the minimum reproducibility / repeatability was 95% (with lower limit equal to 90%).

Required sample sizes

Fig 6 provides an overview of the power as a function of the number of slides for the different values of reproducibility/repeatability for each level of repeatability/reproducibility separately. Given that we are testing both repeatability and reproducibility at same time for each process, and that we are testing for non-inferiority (at least 99%), we set the level of significance at 0.1/2. Based on these assumptions the, required KK thick smears that need to be re-processed equals 90 for each of the three hypotheses.

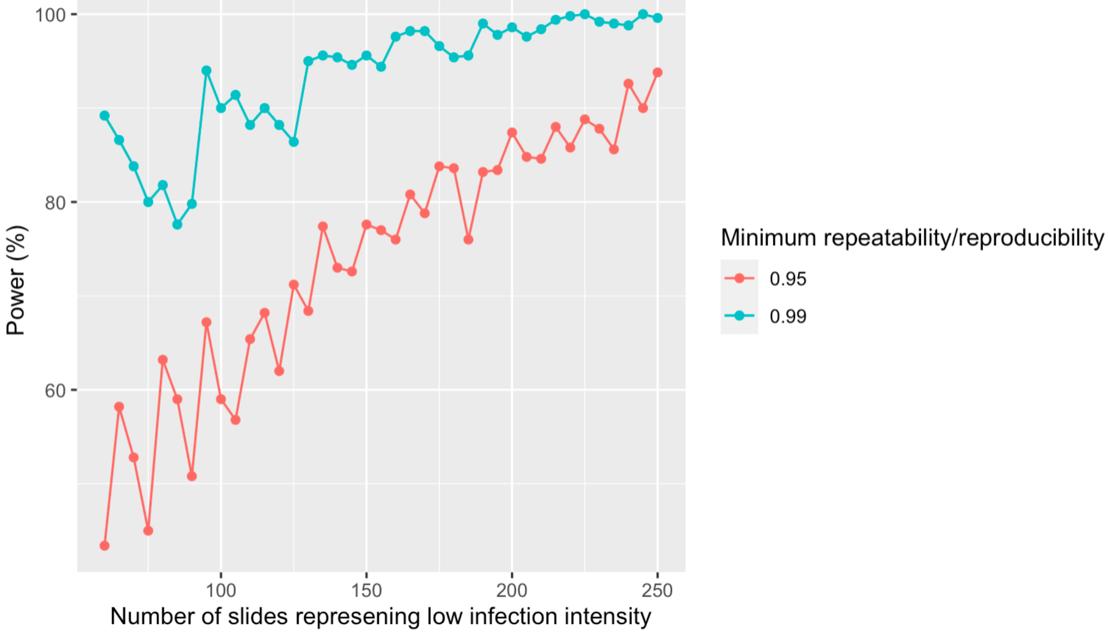

Fig 6. The power to the hypothesis that reproducibility/repeatability KK2.0 is at least 95% and 99%.

R-Markdown code

````
---
title: "Appendix Supplementary Info"
output:
  word_document: default
  pdf_document: default
  html_document: default
---


## 1. Background

This is an R Markdown document that supports the Supplementary Info of the study protocol 'Comprehensive evaluation of an artificial intelligence based digital platform to monitor large-scale deworming programs against soil-transmitted helminthiases: a study protocol' by Ward et al. It is added to provide full insights into the methodology with the ultimate aim to allow researchers both to reproduce and adapt the source code to own needs. To fully align with the Supplementary Info and the main document, we have also structured this document on an experiment basis.

## 2. Experiment 1: Diagnostic performance
### 2.1. Clinical sensitivity to detect low intensity Ascaris, Trichuris and hookworm infections
#### 2.1.1. Functions
```{r result, echo=TRUE}
# function to generate test results (1: positive; 0: negative) assuming that one egg will be detected with a probability px when it is presented to a human eye/AI.
result <- function (N_tot,p_x,n_eggs) rbinom(N_tot,1,prob=1 - (1 - p_x)^n_eggs)
result(10,0.5,1:10) # an example
```

```{r figure2, echo=TRUE}
# figure 2
library(ggplot2)
p_x <- c(0.5,0.6,0.7,0.8,0.9,0.95)
n_i <- seq(1,10,1)
data <- expand.grid(p_x = p_x,n_i= n_i)
data$pi_xi <- 1 - (1 - data$p_x)^data$n_i
ggplot(data = data, aes(x=n_i, y=pi_xi, group=factor(p_x))) +
  geom_line(aes(color=factor(p_x)))+
  geom_point(aes(color=factor(p_x))) +
  guides(color = guide_legend(title = expression(italic(p[x])))) +
  labs(y = expression(paste(pi[ix]^'+')), x = expression(italic(n[i])))
```


```{r dataset, echo=TRUE}
````
````

````
# function to generate a data set of an experiment during which a random set of N_tot KK smears with maximum n_max eggs are screened twice - once with test 1 that detects one egg with probability pi_1, and once with test 2 that detects one egg with a probability pi_2
dataset <- function (N_tot,n_max,p_1,p_2) {
  n_gt = sample(c(1:n_max), N_tot, replace = TRUE)
  data <- data.frame(id = c(1:N_tot),test1 = result(N_tot,p_1,n_gt), test2 = result(N_tot,p_2,n_gt), n_gt = n_gt)
  return(data)
}
dataset(10,10,0.5,0.5) # an example - Table 1
```

```{r check, echo=TRUE}
# function to test for non-inferiority based on confidence intervals
check <- function (ll,t)
  {ifelse(ll >= t,1,0) }
check(-0.5,-0.5) # an example
```

```{r power, echo=TRUE}
library(misty)
# function to determine the power to test the hypothesis that the clinical sensitivity of test 1 is non-inferior compared to that of test 2. The level of equivalence was set at t and the type I error was set at alpha.
power <- function (N_tot,n_max,p_1,p_2,iter,alpha,t) {
  count <- NULL
  for (i in 1:iter)  {
    data <- dataset(N_tot,n_max,p_1,p_2)
    count[i] <- check(ci.prop.diff(data$test1,data$test2, paired=TRUE, conf.level = (1-alpha),method='newcombe', output=FALSE)$result[7],t)
  }
  prop <- sum(count)/iter
  return(prop)
}
power(N_tot=90,n_max=208,p_1=1,p_2=1,iter=1,alpha=0.1/3,t=-0.05) # an example
```

#### 2.1.2. Simulation study
```{r para, echo=TRUE}
# defining the values for each of the parameters
par_grid <- expand.grid(n_max = c(208,41,83),p_1 = c(0.5,0.6,0.7,0.8,0.9,0.95,1),p_2 = c(0.5,0.6,0.7,0.8,0.9,0.95,1), N_tot = seq(60,250,5))
par_grid$helminth <- ifelse(par_grid$n_max == 208, 'Ascaris', ifelse(par_grid$n_max==41, 'Trichuris', 'Hookworm'))
par_grid <- subset(par_grid, par_grid$p_1==par_grid$p_2)
dim(par_grid)
par_grid[1:10,]
````

````
```

```{r simul, echo=TRUE}
# data generation process
for (j in 1:length(par_grid$N_tot))  {
  par_grid$pow[j] <- power(N_tot=par_grid$N_tot[j],
n_max=par_grid$n_max[j],p_1=par_grid$p_1[j],p_2=par_grid$p_2[j],iter=500,t=-0.05,
alpha=0.1/3)
}
```

```{r analyse, echo=TRUE}
# figure 3
library(ggplot2)
ggplot(data = par_grid, aes(x=N_tot, y=100*pow, group=factor(p_1))) +
  geom_line(aes(color=factor(p_1))) +
  geom_point(aes(color=factor(p_1))) +
  labs(x = "Number of KK thick smears representing low intensity infections", y = "Power (%)")
+
  guides(color = guide_legend(title = expression(italic(p[x])))) +
  facet_wrap(~ helminth)
```

```{r sample, echo=TRUE}
# Table 3
library(plyr)
par_grid$pow2 <- ifelse(par_grid$pow<0.80,0,1)
n_max = c(208,41,83)
p_1 = c(0.5,0.6,0.7,0.8,0.9,0.95,1)
n <- length(n_max)*length(p_1)
set4 <- data.frame(n_max=rep(NA,n),p_1=rep(NA,n),sample.size=rep(NA,n))
k<-1
for (h in 1:length(p_1))  {
   set <- subset(par_grid, par_grid$p_1 == p_1[h])
    for (i in 1:length(n_max))  {
      set2 <- subset(set, set$n_max == n_max[i])
      for (j in 1:length(set2$N_tot))  {
      set2$check[j] <-
sum(set2$pow2[j],set2$pow2[j+1],set2$pow2[j+2],set2$pow2[j+3],set2$pow2[j+4],
na.rm=TRUE)
      }
      set3 <- subset(set2,set2$check==5)
      set4$n_max[k] <- n_max[i]
      set4$p_1[k] <- p_1[h]
      set4$sample.size[k] <- min(set3$N_tot)
      k <- k + 1
    }
````

 }
set4
```

### 2.2. Clinical sensitivity to detect moderate-to-heavy intensity Ascaris, Trichuris and hookworm infections
#### 2.2.1. Functions
```{r egg_counts, echo=TRUE}
# function to generate N_tot egg counts when assuming that the counting process follows a binomial distribution with probability of succcess equal to p_x and n_eggs Bernouilli trials.
egg_counts <- function (N_tot,p_x,n_eggs) {rbinom(N_tot,n_eggs,p_x)
}
egg_counts(100,0.2,1:100) # an example
```

```{r dataset2, echo=TRUE}
# function to generate a data set of an experiment during which a random set of N_tot KK thick smears with true egg counts between eggs between n_max_low and n_max_mod are screened twice - once with test 1 that detects eggs with probability pi_1, and once with test 2 that detects eggs with a probability pi_2
dataset2 <- function (N_tot,n_max_low,n_max_mod,p_1,p_2) {
  gt = sample(c((n_max_low+1):n_max_mod), N_tot, replace = TRUE)
  data <- data.frame(id = rep(1:N_tot),gt = gt,test1 = egg_counts(N_tot,p_1,gt), test2 = egg_counts(N_tot,p_2,gt))
  data$mhi_1 <- ifelse(data$test1 > n_max_low, 1, 0)
  data$mhi_2 <- ifelse(data$test2 > n_max_low, 1, 0)
  return(data)
}
dataset2(10,10,100,0.5,0.5) # an example
```

```{r power_mhi, echo=TRUE}
# function to determine the power to test the hypothesis that the clinical sensitivity to detect moderate-to-heavy intensity infections of test 1 is non-inferior compared to that of test 2. The level of equivalence was set at t and the type I error was set at alpha.
power_mhi <- function (N_tot,n_max_low,n_max_mod,p_1,p_2,iter,alpha,t) {
  count <- NULL
  for (i in 1:iter)  {
    data <- dataset2(N_tot,n_max_low,n_max_mod,p_1,p_2)
    count[i] <- check(ci.prop.diff(data$mhi_1,data$mhi_2, paired=TRUE, conf.level = (1-alpha),method='newcombe', output=FALSE)$result[7],t)
  }
  prop <- sum(count)/iter
  return(prop)
}
power_mhi(N_tot=90,n_max_low=10,n_max_mod=100,p_1=0.2,p_2=0.2,iter=10,alpha=0.05,t=-0.05) # an example

````
```

2.2.2. Simulation study
```{r para2, echo=TRUE}
# defining the values for each of the parameters
par_grid <- expand.grid(n_max_low = c(208,41,83),p_1 = c(0.5,0.6,0.7,0.8,0.9,0.95),p_2 =
c(0.5,0.6,0.7,0.8,0.9,0.95), N_tot = seq(100,350,5))
par_grid <- subset(par_grid, par_grid$p_1==par_grid$p_2)
par_grid$helminth <- ifelse(par_grid$n_max_low == 208, 'Ascaris',
ifelse(par_grid$n_max_low==41, 'Trichuris', 'Hookworm'))
par_grid$n_max_mod <- ifelse(par_grid$n_max_low == 208, 2083,
ifelse(par_grid$n_max_low==41, 416, 166))
dim(par_grid)
par_grid[1:10,]
```

```{r simul2, echo=TRUE}
# data generation process
for (j in 1:length(par_grid$N_tot)) {
  par_grid$pow[j] <-
power_mhi(N_tot=par_grid$N_tot[j],n_max_low=par_grid$n_max_low[j],n_max_mod=par_
grid$n_max_mod[j],p_1=par_grid$p_1[j],p_2=par_grid$p_2[j],iter=500,alpha=0.10/3,t=-
0.05)
}
```

```{r analyse2, echo=TRUE}
# figure 4
library(ggplot2)
ggplot(data = par_grid, aes(x=N_tot, y=100*pow, group=factor(p_1))) +
  geom_line(aes(color=factor(p_1))) +
  geom_point(aes(color=factor(p_1))) +
  labs(x = "Number of KK thick smears represening MHI infections", y = "Power (%)") +
  guides(color = guide_legend(title = expression(italic(p[x])))) +
  facet_wrap(~ helminth)
```

```{r sample2, echo=TRUE}
# Table 5
library(plyr)
par_grid$pow2 <- ifelse(par_grid$pow<0.80,0,1)
n_max_low = c(208,41,83)
p_1 = c(0.5,0.6,0.7,0.8,0.9,0.95)
n <- length(n_max_low)*length(p_1)
set4 <- data.frame(n_max_low=rep(NA,n),p_1=rep(NA,n),sample.size=rep(NA,n))
h<- 1
i <- 1
````

```
k<-1
for (h in 1:length(p_1))  {
  p_10 <- p_1[h]
   set <- subset(par_grid, par_grid$p_1 == p_10)
    for (i in 1:length(n_max_low))  {
      set2 <- subset(set, set$n_max_low == n_max_low[i])
      for (j in 1:length(set2$N_tot))  {
      set2$check[j] <-
sum(set2$pow2[j],set2$pow2[j+1],set2$pow2[j+2],set2$pow2[j+3],set2$pow2[j+4],
na.rm=TRUE)
      }
      set3 <- subset(set2,set2$check==5)
      set4$n_max_low[k] <- n_max_low[i]
      set4$p_1[k] <- p_1[h]
      set4$sample.size[k] <- min(set3$N_tot)
      k <- k + 1
    }
 }
set4
```

2.3. Clinical specificity to detect any and moderate-to-heavy intenstity Ascaris, Trichuris and hookworm infections
2.3.1. Functions
```{r p, echo=TRUE}
# function to generate the probabilities p in each cell of a 2x2 table representing the test results of 2 tests on negative slides when the specificity equals sp across varying levels of concordance in false positive test results (level 1: no concordance; level 2: moderate; level 3: perfect)
p <- function (sp,level) {
  if(level==3) {
    p_a = 1-sp
    p_b = 0
    p_c = 0
    p_d = sp
  }
  else if (level == 2) {
    p_a = (1-sp)/3
    p_b = (1-sp)/3
    p_c = (1-sp)/3
    p_d = sp
  } else {
    p_a = 0; p_b = (1-sp)/2; p_c = (1-sp)/2; p_d= sp
  }
  return(c(p_a,p_b,p_c,p_d))
}
```

````
p(0.95,level=3) # an example
matrix(rmultinom(1,10,p(0.50,level=1)), nrow=2, byrow=TRUE) # an operational example
```

```{r dataset_sp, echo=TRUE}
# function to generate a data set based on a 2x2 table representing the test results of 2 tests on negative slides when the de specificity equals sp across varying levels of concordance in false positive test results (level 1: no concordance; level 2: moderate; level 3: perfect)
dataset_sp <- function (matrix) {
  data <- data.frame(id = rep(1:sum(matrix)),
            test1 = c(rep(1,matrix[1,1]),rep(0,matrix[1,2]),rep(1,matrix[2,1]),rep(0,matrix[2,2])),
            test2 = c(rep(1,matrix[1,1]),rep(1,matrix[1,2]),rep(0,matrix[2,1]),rep(0,matrix[2,2])))
  return(data)
}
mat <- matrix(rmultinom(1,10,p(0.50,level=1)), nrow=2, byrow=TRUE) # check 1
data <- dataset_sp(mat) # check 2
table(data$test1,data$test2) # check 3
```

```{r power_sp, echo=TRUE}
# function to determine the power to test the hypothesis that the clinical specificity to detect any or moderate-to-heavy intensity infections of test 1 is non-inferior compared to that of test 2. The level of equivalence was set at t and the type I error was set at alpha.
power_sp <- function (N_tot,sp,level,iter,alpha,t) {
  count <- NULL
  for (i in 1:iter)  {
    mat <- matrix(rmultinom(1,N_tot,p(sp,level)), nrow=2,byrow=TRUE)
    data <- dataset_sp(mat)
    count[i] <- check(ci.prop.diff(data$test1,data$test2, paired=TRUE, conf.level = (1-alpha),method='newcombe', output=FALSE)$result[7],t)
  }
  prop <- sum(count)/iter
  return(prop)
}
power_sp(N_tot=100,sp=0.99,level=2,iter=5,alpha=0.05/3,t=-0.05) # an example
```
#### 2.2.2. Simulation study
```{r para3, echo=TRUE}
# Values for different parameters
par_grid <- expand.grid(N_tot = seq(100,350,5), sp = seq(0.9,1,0.01),level=c(1,2,3))
dim(par_grid)
```

```{r simul3, echo=TRUE}
for (j in 1:length(par_grid$N_tot))  {
````

~~~
  par_grid$pow[j] <- power_sp(N_tot=par_grid$N_tot[j],
sp=par_grid$sp[j],level=par_grid$level[j],iter=500,t=-0.05, alpha=0.05/3)
}
```

```{r analyse3, echo=TRUE}
# Figure 5
ggplot(data = par_grid, aes(x=N_tot, y=100*pow, group=factor(sp))) +
  geom_line(aes(color=factor(sp))) +
  geom_point(aes(color=factor(sp))) +
  labs(x = "Number of KK smears represening no/low intensity intensity", y = "Power (%)") +
  guides(color = guide_legend(title = 'Specificity'))+
  facet_wrap(~ factor(level))
```

```{r sample3, echo=TRUE}
# Table 8
library(plyr)
par_grid$pow2 <- ifelse(par_grid$pow<0.80,0,1)
sp <-  seq(0.9,1,0.01)
level <- c(1,2,3)
n <- length(sp)*length(level)
set4 <- data.frame(sp=rep(NA,n),level=rep(NA,n),sample.size=rep(NA,n))
h<- 2
i <- 2
k<-1
for (h in 1:length(sp))  {
  sp0 <- sp[h]
   set <- subset(par_grid, par_grid$sp == sp0 )
    for (i in 1:length(level))  {
      set2 <- subset(set, set$level == i)
      for (j in 1:length(set2$N_tot))  {
      set2$check[j] <-
sum(set2$pow2[j],set2$pow2[j+1],set2$pow2[j+2],set2$pow2[j+3],set2$pow2[j+4],
na.rm=TRUE)
      }
      set3 <- subset(set2,set2$check==5)
      set4$sp[k] <- sp0
      set4$level[k] <- level[i]
      set4$sample.size[k] <- min(set3$N_tot)
      k <- k + 1
    }
 }
set4
```
## 3. Work package 2: Reproducibility
#### 3.1. Functions
~~~

````
```{r power_rep, echo=TRUE}
library(Hmisc)
# function to determine the power to test the hypothesis that the reproducibility/repeatability of test results is at least t.
power_rep <- function (N_tot,alpha,iter,pi,t)
{
  count <- NULL
  for (i in 1:iter)  {
  x <- rbinom(1,N_tot, prob = pi)
    count[i] <- check(binconf(x, N_tot, alpha=alpha, method=c("asymptotic"))[1,2],t)
  }
    prop <- sum(count)/iter
  return(prop)
}
power_rep(140,0.1,500,0.95,0.90) # an example
```

#### 3.2. Simulation
```{r para4, echo=TRUE}
# Values for different parameters
par_grid <- expand.grid(N_tot = seq(60,250,5), pi = c(0.95,0.99), alpha = c(0.1), ntests = c(2))
par_grid$t <- ifelse(par_grid$pi==0.95,0.9,0.95)
dim(par_grid)
```

```{r simul4, echo=TRUE}
for (j in 1:length(par_grid$N_tot))  {
  par_grid$pow[j] <- power_rep(N_tot=par_grid$N_tot[j],alpha=(par_grid$alpha[j]/par_grid$ntests[j]), iter=500,pi=par_grid$pi[j],t=par_grid$t[j])
}
```

```{r analyse4, echo=TRUE}
# Figure 6
library(ggplot2)
ggplot(data = par_grid, aes(x=N_tot, y=100*pow, group=factor(pi))) +
  geom_line(aes(color=factor(pi))) +
  geom_point(aes(color=factor(pi))) +
  labs(x = "Number of slides represening low infection intensity", y = "Power (%)") +
  guides(color = guide_legend(title = 'Minimum repeatability/reproducibility'))+
  facet_wrap(~ factor(ntests))
```

```{r sample4, echo=TRUE}
# Table 9
library(plyr)
````
````

```
par_grid$pow2 <- ifelse(par_grid$pow<0.80,0,1)
pi = c(0.95,0.99)
ntests = c(1,2,3,4)
n <- length(pi)*length(ntests)
set4 <- data.frame(pi=rep(NA,n),ntests=rep(NA,n),sample.size=rep(NA,n))
h<- 2
i <- 1
k<-1
for (h in 1:length(pi))  {
  pi0 <- pi[h]
   set <- subset(par_grid, par_grid$pi ==  pi0 )
    for (i in 1:length(ntests))  {
      set2 <- subset(set, set$ntests == i)
      for (j in 1:length(set2$N_tot))  {
      set2$check[j]                                                                    <-
sum(set2$pow2[j],set2$pow2[j+1],set2$pow2[j+2],set2$pow2[j+3],set2$pow2[j+4],
na.rm=TRUE)
      }
      set3 <- subset(set2,set2$check==5)
      set4$pi[k] <-  pi0
      set4$ntests[k] <- ntests[i]
      set4$sample.size[k] <- min(set3$N_tot)
      k <- k + 1
    }
 }
set4
```
```

## References


1. Cools P, Vlaminck J, Albonico M, Ame S, Ayana M, José Antonio BP, et al. Diagnostic performance of a single and duplicate Kato-Katz, Mini-FLOTAC, FECPAKG2 and qPCR for the detection and quantification of soil-transmitted helminths in three endemic countries. Plos Neglect Trop D. 2019;13(8):e0007446. doi: 10.1371/journal.pntd.0007446.
2. Newcombe RG. Improved confidence intervals for the difference between binomial proportions based on paired data. Stat Med. 1998;17(22):2635-50. Epub 1998/12/05. PubMed PMID: 9839354.
3. Montresor A, Crompton DWT, Hall A, Bundy DAP, Savioli L, World Health Organization. Division of Control of Tropical Diseases S, et al. Guidelines for the evaluation of soil-transmitted helminthiasis and schistosomiasis at community level : a guide for managers of control programmes / A. Montresor ... [et al.]. Geneva: World Health Organization; 1998.
4. Vlaminck J, Cools P, Albonico M, Ame S, Ayana M, Dana D, et al. An in-depth report of quality control on Kato-Katz and data entry in four clinical trials evaluating the efficacy of albendazole against soil-transmitted helminth infections. PLoS Negl Trop Dis. 2020;14(9):e0008625. Epub 2020/09/22. doi: 10.1371/journal.pntd.0008625. PubMed PMID: 32956390; PubMed Central PMCID: PMCPMC7549791.
```
