# Supplementary material for: A comprehensive evaluation of an artificial intelligence based digital pathology to monitor large-scale deworming programs against soil-transmitted helminths: A study protocol
Source: PLoS One. 2024 Oct 28;19(10):e0309816. doi: 10.1371/journal.pone.0309816 (PMC11515989; doi:10.1371/journal.pone.0309816)
Supplement: S5 File — (PDF) [file pone.0309816.s005.pdf]

EVALUATION OF AN AI-DP FOR STH DEWORMING PROGRAMS

A Data Management Plan created using DMPonline.be

Creators: Peter Ward, Bruno Levecke https://orcid.org/https://orcid.org/0000-0001-8912-5595, n.n. n.n.

Affiliation: Ghent University (UGent - UZ Gent)

Template: Horizon Europe DMP +

Grant number / URL: 76906491, J&J Foundation

ID: EN-2023-CT001

Start date: 01-10-2023

End date: 31-06-2024

Project abstract:
Manual screening of a Kato-Katz (KK) thick stool smear remains the current standard to monitor the impact of large-scale deworming programs against soil-transmitted helminths (STHs). To improve this diagnostic standard, we recently designed an artificial intelligence based digital pathology system (AI-DP) for digital image capture and analysis of KK thick smears. Preliminary results of its diagnostic performance are encouraging, and a comprehensive evaluation of our AI-DP as a cost-efficient end-to-end diagnostic to inform STHs control programs against the target product profiles (TPP) of the World Health Organisation (WHO) is the next step for validation.

Last modified: 07-09-2023

KAKDU: EVALUATION OF AN AI-DP FOR STH DEWORMING PROGRAMS

Full DMP

VERSION INFORMATION

Action number

EN-2023-CT001

Action acronym

KAKADU

Action title

Evaluation of an AI-DP for STH deworming programs

DMP version number

V1.0

Creation Date

2nd September 2023

1. DATA SUMMARY

1.1 Will you re-use any existing data and what will you re-use it for?

Yes, we intend to reuse existing data in our project. Specifically, we plan to reuse data from the Starworms project (starworms.org; https://doi.org/10.1371/journal.pntd.0011071) for the following purposes:

General Framework and Cost-Efficiency Analysis: We will utilize the general framework and cost-efficiency analysis data from the Starworms project to support cost-efficient study design choices for large-scale soil-transmitted helminth (STH) deworming programs. This data will aid in comparing the cost-effectiveness of our AI-based diagnostic approach with traditional methods.

Itemized Cost-Assessment: We will reuse itemized cost-assessment data from the Starworms project to inform our own cost-efficiency analysis for the AI-DP-based deworming program evaluation.

Cost-Efficiency Data: The data on cost-efficiency, including various parameters such as the number of stool samples, number of KK smears, operational settings, diagnostic platforms, and other factors, will be drawn from the Starworms project to provide a basis for cost-effectiveness comparisons.
Reusing this existing data is cost-effective and aligns with the objectives of our project. It allows us to build upon prior research efforts and leverage valuable insights and cost-related information from the Starworms project. We have ensured compliance with any regulatory or contractual restrictions regarding data reuse and sharing, including any confidentiality agreements in place.

AI Models and Training Data: We plan to include the reuse of existing scan data and AI models from past projects, specifically the AI4NTD consortium (www.ai4ntd.org). These data and models will be valuable for our project as they can provide a foundation for training and evaluating AI models for the identification of worm eggs in stool samples. Reusing these resources will help accelerate our research and leverage the expertise and insights gained from previous projects in the field of neglected tropical diseases (NTDs).

1.2 What types and formats of data and other research outputs will the project generate or re-use?

We will comprehensively evaluate an artificial intelligence based digital pathology (AI-DP) based on its (i) diagnostic performance, (ii) repeatability/reproducibility, (iii) time-to-result, (iv) cost-efficiency to inform large-scale deworming programs and (v) usability in both laboratory and field settings. For each of these five attributes, we designed separate experiments with sufficient power to verify the non-inferiority of the AI-DP (KK2.0) over the current diagnostic standard, namely the manual counting worm eggs in KK thick smear (KK1.0). These experiments will be conducted in two STH endemic countries with national deworming programs (Ethiopia and Uganda), focusing on school-age children (SAC) only. In the following sections we will indicate what type and formats of data will be generated / re-used by the project.

(I) Diagnostic performance

- **School characteristics:** school name, unique school ID, administrative level of school location, country, school geocoordinates; data will be collected through an electronic data capture tool (EDC; Zoho Creator); data will be in the form of SQL and xls.

- **Participant consent and assent:** participants full name, guardian consent, consent/assent from participant, date and signature; data will be collected on paper only (Participation Consent Form) and will be stored in country (Ethiopia and Uganda).

- **Participant registration:** full name of participants, participant ID, name school, school ID, sex, age (in years), grade and status of consent; interviewer initials; type of treatment; data will be collected on paper only (Participant Register Form) and will be stored in country (Ethiopia and Uganda)

- **Participant demographics:** school ID; participant ID, sex, age (in years) and status of consent; data will be collected through an EDC (Zoho Creator) based on the Participant Register form; data will be in the form of SQL and xls.

- **Slide preparation:** participant ID, Bristol stool index, time stamps when KK thick smear for this subject ID was prepared; data will be collected through an electronic data capture tool (EDC; Zoho Creator) by scanning unique QR code on slide; data will be in the form of SQL and xls.

- **KK1.0 results:** date of analysis; participant ID; number of eggs manually counted for each worm species; date of laboratory analysis; initials of laboratory technician; data will be collected on paper (KK1.0 Form); afterwards data will be digitized through an electronic data capture tool (EDC; Zoho Creator) by scanning unique QR code on slide; data will be in the form of SQL and xls.

- **Scans of Whole Slide Imager:** images (.jpeg) with metadata containing scan information (e.g., scan time, scan ID, participant ID, AI-identified objects); data will be in the form of SQL.

- **Raw KK2.0 results:** participant ID; study phase, random allocation identifier; number of AI-identified objects classified as *Ascaris*, *Trichuris*, hookworms, *Schistosoma mansoni* or other worms; data will be generated in EggInspector and will be in the form of SQL and xls.

- **Verified KK2.0 results:** participant ID; time to verify images; number of AI-identified objects verified as *Ascaris*, *Trichuris*, hookworms, *S.mansoni* or other worms; data will be generated in EggInspector and will be in the form of SQL and xls.

- **Ground truth results:** participant ID; number of confirmed *Ascaris*, *Trichuris*, hookworms, *Schistosoma mansoni* or other worms; data will be generated in EggInspector data and will be in the form of SQL and xls.

- **AI models** developed with data generated in the study for object identification in scanner image; the AI model will be in the form of ONNX files.

(ii) Repeatability/reproducibility

- **KK1.0 results:** date of analysis; slide ID; random allocation identifier; number of eggs manually counted for each worm species; date of laboratory analysis; initials of laboratory technician; data will be collected on paper (KK1.0 Form); afterwards data will be digitized through an electronic data capture tool (EDC; Zoho Creator) by scanning unique QR code on slide; data will be in the form of SQL and xls.

- **Scans of Whole Slide Imager:** images (.jpeg) with metadata containing scan information (e.g., scan time, scan ID, slide ID, AI-identified objects); data will be in the form of SQL and xls.

- **Raw KK2.0 results:** participant ID; random allocation identifier; number of AI-identified objects classified as Ascaris, Trichuris, hookworms, Schistosoma mansoni or other worms; data will be generated in EggInspector and will be in the form of SQL and xls.

- **Verified KK2.0 results:** participant ID; time to verify images; number of AI-identified objects verified as Ascaris, Trichuris, hookworms, Schistosoma mansoni or other worms; data will be generated in EggInspector and will be in the form of SQL and xls.

(iii) time-to-result

- Time stamps of both scanning and annotating will be part of the diagnostic performance (see Raw KK2.0 results; Verified KK2.0 results)

(iv) cost-efficiency to inform large-scale deworming programs

- **General framework:** we will update two general frameworks that were previously developed to support cost-efficient study design choices for large-scale STH deworming programs; we will re-use some of the data collected as part of the Starworms project (starworms.org; https://doi.org/10.1371/journal.pntd.0011071); data/code will be in the form of an R-file.

- **Itemized cost-assessment:** item, amount/number, cost per unit in US$, life expectancy in years/how many samples used, cost per individual sample in US$ accounting for life expectancy, total cost per sample.

- **Cost-efficiency:** number of stool samples, number of Kato-Katz slides, operational setting, diagnostic platform, level of endemicity, program threshold, probability of overtreatment, probability of undertreatment, worm species, total survey cost; data will be in the form of an xls and txt.

(v) usability in both laboratory and field settings.

- **Interviews:** audio- and video records of interviews; data will be in the form of mp3 and mp4.

- **Verbatim transcription:** profile interviewee, transcriptions of interview; data will be in the form of word and pdf documents.

1.3 What is the purpose of the data generation or re-use and its relation to the objectives of the project?

The purpose of the data generation is to comprehensively evaluate an AI-DP as a cost-efficient end-to-end diagnostic to inform STH against the target product profiles of the World Health Organization. To this end, we are collecting data (i) diagnostic performance, (ii) repeatability/reproducibility, (iii) time-to-result, (iv) cost-efficiency to inform large-scale deworming programs and (v) usability in both laboratory and field settings.

1.4 What is the expected size of the data that you intend to generate or re-use?

A total of 1,100 school-aged children (SAC; 600 in Ethiopia and 500 in Uganda) will be enrolled in this study. For each subject, one Kato-Katz thick smear will be prepared, which will be screened both manually (KK1.0) and by the AI-DP (KK2.0). A subset of the samples (90 KK thick smear) will be re-analysed to assess the repeatability/reproducibility. It is anticipated that the entire size of data is approximately 1.5 TB. The digitalised scans (1GB each; 1500 in total) will account for most data (99.997%).

1.5 What is the origin/provenance of the data, either generated or re-used?

The stool will be collected from SAC in Ethiopia and Uganda. Data on the five parameters will be generated by field/laboratory workers, engineers and researchers in Belgium, Ethiopia, Sweden and Uganda.

1.6 To whom might your data be useful ('data utility'), outside your project?

The data will be useful for health decision makers (e.g., Ministries of Health of STH-endemic countries, WHO), researchers and global health experts in the field of neglected tropical diseases and AI, and international funding bodies supporting the control of neglected tropical diseases (e.g., Bill & Melinda Gates Foundation, END Fund, USAID, Childrens Investment Fund, Children Without Worms, …).

2.1 FAIR DATA: MAKING DATA FINDABLE, INCLUDING PROVISIONS FOR METADATA

2.1.1 Will data and other research outputs be identified by a persistent identifier?

- Yes: describe below

Yes, DOI numbers will be assigned to research outputs. Any data made public via relevant data repositories will be assigned an accession number.

2.1.2 Will rich metadata be provided to allow discovery?
What metadata will be created?
What disciplinary or general standards will be followed?
In case metadata standards do not exist in your discipline, please outline what type of metadata will be created and how.

(I) Diagnostic performance

- Worm specific egg count data obtained by manual egg counting and AI-DP (raw, verified and ground truth) will be supplemented with demographic data of the participants (participant demographics), school characteristics and Bristol stool index.

- Image datasets will contain annotated labels of images (worm species, location within image)

(ii) Repeatability/reproducibility

Image datasets will contain the following metadata: annotated labels of images (worm species, location within image)
Study results will include the following metadata: egg count, infection intensity (based on WHO categories), AI performance (precision/recall), clinical diagnostic performance (sensitivity/specificity) for each worm species separately.
The system will also be assessed and provide data relating to cost-efficiency analysis results, and useability reporting.

2.1.3 Will search keywords be provided in the metadata to optimize the possibility for discovery and then potential re-use?

- Yes: describe below

Yes, standard and searchable metadata headings will be used.

2.1.4 Will metadata be offered in such a way that it can be harvested and indexed?

- Yes: describe below

Yes, and publications associated with study outputs will provide indexing on search engines and common journal repositories (e.g., PubMed, Google Scholar).

2.2 FAIR DATA: MAKING DATA ACCESSIBLE

2.2.1 Will the data and other research outputs be deposited in a trusted repository?

- Yes: describe below

Open access journals that are known and reputable in the field of parasitology and diagnostics will be used for publications (e.g., PLoS Neglected Tropical Diseases). Any published datasets will be uploaded to reliable data repositories (e.g., www.kaggle.com).

2.2.2 Have you explored appropriate arrangements with the identified repository where your data and other research outputs will be deposited?

- Yes

The study team has previous experience in sharing data from similar studies using www.kaggle.com (https://doi.org/10.1371/journal.pntd.0010500) or as a supplementary info of publications (https://doi.org/10.1371/journal.pntd.0007471.s004)

2.2.3 Does the repository ensure that the data and other research outputs are assigned an identifier? Will the repository resolve the identifier to a digital object?

Yes, Kaggle provides a unique DOI citation for each uploaded dataset. This is also the case for original datasets submitted to open access peer reviewed journals such as PLoS NTDs (https://doi.org/10.1371/journal.pntd.0007471.s004)

2.2.4 Will all data and other research outputs be made openly available?

- Yes

Results of this comprehensive validation (e.g., diagnostic performance, repeatability/reproducibility, cost-efficiency and usability) will be made available under open access.
All images will be available upon request at completion of the project under Creative Commons Attribution-ShareAlike 4.0 International (CC BY-SA 4.0) license. Within 1 year of completion of the project, a relevant selection of the image database will also be made publicly available at a trusted repository with a DOI and tags to make it findable, in combination with final publications on the project.

2.2.5 Is an embargo applied to give time to publish or seek protection of the intellectual property (e.g. patents)?

- Yes

2.2.6 If an embargo is applied (see question 2.2.5), specify why and how long this will apply, bearing in mind that research data should be made available as soon as possible.

Up to 1 year from project completion to publish results. However, data will be available upon request immediately after project completion.

2.2.7 Will the data and other research outputs be accessible through a free and standardized access protocol?

- Yes: describe below

Yes, open access publishers/journals/repositories will be used for any published data.

2.2.8 If there are restrictions on use, how will access be provided to the data, both during and after the end of the project?

For access to full image collection, access will be made available upon request. Access to the published datasets will be available on public repositories with contact information for the full data collection.

2.2.9 How will the identity of the person accessing the data be ascertained?

Open access publications and journal publications are not tracked to individuals downloading the article, to the best of our knowledge. Similarly, any data downloaded from a repository may not track the individual downloading that dataset.

2.2.10 Is there a need for a data access committee (e.g., to evaluate/approve access requests to personal/sensitive data)?

- No

No, data that will become publicly available will be anonymised.

2.2.11 Will metadata be made openly available and licenced under a public domain dedication CC0, as per the Grant Agreement? If not, please clarify why.

- Yes

Publications and results will be published in open access journals and subject the journals licenses.
Relevant deidentified metadata pertaining to the image database for training AI models will be available under Attribution-ShareAlike 4.0 International (CC BY-SA 4.0).

2.2.12 Will metadata contain information to enable the user to access the data?

- No

2.2.13 How long will the data remain available and findable? Will metadata be guaranteed to remain available after data is no longer available?

Data will be made publicly available through trusted data repositories or as supplementary info of publications. See above.

2.2.14 Will documentation or reference about any software needed to access or read the data be included? Will it be possible to include the relevant software (e.g., in open-source code)?

No software will be needed to access study results.

2.3 FAIR DATA: MAKING DATA INTEROPERABLE

2.3.1
What data and metadata vocabularies, standards, formats or methodologies will you follow to make your data interoperable to allow data exchange and re-use within and across disciplines?
Will you follow community-endorsed interoperability best practices? Which ones?

Results will be published in common and open data formats such as pdf, doc, xls, txt, json, csv, jpegs, or TFRECORDS formats.

2.3.2 In case it is unavoidable that you use uncommon or generate project specific ontologies or vocabularies:
Will you provide mappings to more commonly used ontologies?
Will you openly publish the generated ontologies or vocabularies to allow reusing, refining or extending them?

N/A - no new ontologies will be created.

2.3.3 Will your data and other research outputs include qualified references to other data (e.g., other data from your project, or datasets from previous research)?

- Yes

Yes, other publications which will refer to previously used datasets will be noted.

2.4 FAIR DATA: INCREASE DATA RE-USE

2.4.1 How will you provide documentation needed to validate data analysis and facilitate data re-use?

We will publish our detailed study protocol. In addition, all standard operating procedures, and data sets will be made available as supplementary info when published in open access journals.

2.4.2
Will your data and other research outputs be made freely available in the public domain to permit the widest re-use possible?
Will your data and other research outputs be licensed using standard reuse licenses, in line with the obligations set out in the Grant Agreement?

Research outputs will be published in in open access journals and new findings and results will be made available to the general public in layman's terms on the project website and relevant social media platforms as per the projects communications plan.

2.4.3 Will the data and other research output produced in the project be useable by third parties, in particular after the end of the project?

- Yes

Results shared in the form of publications (journals, website, social media) will be freely available for use by third parties under open access journal licences or Creative Commons Attribution-ShareAlike 4.0 International (CC BY-SA 4.0).

2.4.4 Will the provenance of the data and other research outputs be thoroughly documented using the appropriate standards?

- Yes

All raw data generated in the study will have traceable data provenance and documentation.

2.4.5 Describe all relevant data quality assurance processes.

During data analysis, data cleaning will be conducted when datasets are merged and reconciled. Certain metadata will be used to consolidate datasets and ensure wholeness of the data.

3. OTHER RESEARCH OUTPUTS

3.1 Do you have any additional information, that was not addressed in the previous sections, which you wish to provide regarding other research outputs that are generated or re-used throughout the project?

No

4. ALLOCATION OF RESOURCES

4.1 What will the costs be for making data and other research outputs FAIR in your project?

Other than the publication fees, we do not anticipate major costs for making data and other research outputs FAIR in the project.

4.2 How will these be covered?

The publication fees will be covered through the grant budget.

4.3 Who will be responsible for data management in your project?

Enaiblers, Sweden

- Peter Ward, peter.ward@enaiblers.com
- Peter Dahlberg, peter.dahlberg@enaiblers.com
- John Bergelin, john.bergelin@enaiblers.com
- Arya Farhang, arya.farhang@enaiblers.com

Ghent University, Belgium

- Bruno Levecke, bruno.levecke@ugent.be
- Hanne Elsen, privacy@ugent.be

Vector Control Division, Ministry of Health, Uganda

- Betty Nabattae, bettieful@gmail.com
- Narcis Kabatereine, Narcis.Kabatereine@ascend.crownagents.com

Institute of Health, Jimma University, Ethiopia

- Mio Ayana, Kelkiyas@gmail.com
- Zeleke Mekonen, zeleke.mekonnen@ju.edu.et

4.4 How will long term preservation be ensured?

Using reputable and trustworthy data repositories.

5. DATA SECURITY

5.1 What provisions are or will be in place for data security?

Paper records with personal data will be stored in safe locations in VCD and Jimma University. Keys for pseudonymized will not be shared by VCD or Jimma University with any other parties.

Enaiblers EDC app uses encryption for data in transit and at rest.

Publicly shared data will remove data points to break pseudonymization (e.g., school ID, geolocation) and include aggregate so that shared data is anonymous.

5.2 Will the data be safely stored in trusted repositories for long term preservation and curation?

- Yes

Paper records will be stored in secure locations managed by VCD and Jimma University.

Electronic records will be stored encrypted on servers in Europe.

6. ETHICS

6.1 Are there, or could there be, any ethics or legal issues that can have an impact on data sharing?

- No

Data for processing will be pseudonymized, and hence we anticipate no major issues to make the data available. As indicated above. Any data for sharing in public research will be fully anonymized.

6.2 Will informed consent for data sharing and long term preservation be included in questionnaires dealing with personal data?

- Yes

Yes, informed consent will cover data sharing and long-term preservation.

7. OTHER ISSUES

7.1 Do you, or will you, make use of other national/funder/sectorial/departmental procedures for data management? If yes, which ones (please list and briefly describe them)?

- No

KAKDU: EVALUATION OF AN AI-DP FOR STH DEWORMING PROGRAMS

DPIA

DPIA

Have you performed a DPIA for the personal data processing activities for this project?

- Yes

DATA PROTECTION IMPACT ASSESSMENT

1. Does your research fall under the scope of a template DPIA?

- Yes (specify below)

2. Provide more details for any of the risks that you have ticked in question 25 under the 'GDPR record' tab, so the overall risks related to your processing are clearly and accurately described.

1. **Risk of Discrimination and Stigmatization**:
    - Applicability: Children and their families may face discrimination or stigmatization if their participation in the study, especially if they are diagnosed with intestinal worm infections, becomes known within their school or community.
    - Why: Stigmatization or discrimination may occur if other students or community members become aware that a child has a health condition (e.g., worm infection) or if data leakage results in unintended disclosure of health information.
2. **Risk of Data Leaks (Disclosure of Identity or Sensitive Data)**:
    - Applicability: The study involves the collection of personal data, including names, ages, schools, and potentially health-related information, making it susceptible to data leaks.
    - Why: Data leaks could occur due to security breaches, unauthorized access, or accidental disclosure. If the identity or sensitive health information of participants is exposed, it could lead to privacy violations and harm to their reputation.
3. **Risk to the Safety of Participants**:
    - Applicability: Children participating in the study may face safety risks if their personal data, school information, or health status is exposed to unauthorized individuals who may pose a threat.
    - Why: Safety concerns could arise if data leaks result in harmful actions by individuals who gain access to participant information, such as targeting children based on their health conditions or school locations.
4. **Possible Abuse of Research Methodology or Findings**:

- Applicability: The misuse or misinterpretation of research findings could potentially harm participants if the research methodology or findings are used inappropriately.

- Why: If the research findings are misconstrued or misused, it may lead to incorrect conclusions or recommendations, potentially affecting the health and well-being of participants or public health interventions.

5. **Processing of Special Categories of Personal Data**:

   - Applicability: The study involves the processing of special categories of personal data, including health-related information (e.g., stool samples) and data related to children (e.g., age, school).

   - Why: Processing special categories of data, particularly health data related to children, requires enhanced data protection measures due to the sensitivity of this information. Children's data, in particular, necessitates additional safeguards because they are considered a vulnerable group under data protection regulations.

3. Explain why the processing of personal data is necessary to achieve the purposes of the research. Include the benefits for individuals and the wider public.

1. **Diagnosis and Treatment of Intestinal Worm Infections**:

   - Purpose: The primary objective of the research is to diagnose intestinal worm infections in children aged 5-14, and if diagnosed, to provide appropriate treatment.

   - Necessity: Personal data, including names, ages, and schools, are essential for identifying and tracking individual participants. This information is crucial for providing the correct treatment to infected children, improving their overall health, nutritional status, and reducing anaemia.

   - Benefits:

     - Individual Benefits: Children with worm infections will receive timely and effective treatment, improving their health and well-being.

     - Public Benefits: The research contributes to the overall health of the community by reducing the prevalence of worm infections, potentially leading to improved public health outcomes.

2. **Validation of AI-Based Diagnostic Technology**:

   - Purpose: The research aims to validate the use of Artificial Intelligence (AI) for automating the diagnosis of worm infections in stool samples.

   - Necessity: Personal data, including stool samples and associated participant information, are required to train and evaluate AI models accurately. This data is vital for developing a reliable diagnostic tool.

   - Benefits:

     - Individual Benefits: Participants benefit from accurate and efficient diagnostic methods, reducing the time and effort required for diagnosis.

     - Public Benefits: The research contributes to the development of advanced diagnostic technologies, which can enhance healthcare services and potentially lead to more accessible and cost-effective diagnostics for intestinal worm infections on a global scale.

3. **Public Health Improvement and Sustainability of Deworming Programs**:

    - Purpose: The research contributes to public health by assessing the prevalence of intestinal worm infections in school-age children and the effectiveness of deworming programs.

    - Necessity: Personal data enable researchers to accurately assess infection rates and the impact of interventions. This data informs public health policies and program sustainability.

    - Benefits:

        - Individual Benefits: Participants indirectly benefit from improved public health outcomes, as the research helps identify areas where interventions are needed and ensures program effectiveness.

        - Public Benefits: The findings of the research can guide policymakers, leading to more effective and sustainable deworming programs that benefit entire communities and populations, especially in regions where these infections are prevalent.

4. Can the processing or part of the processing reasonably be achieved in a different/alternative way, less detrimental to the privacy of data subjects? Evaluate the possible alternatives.

- No

The processing of personal data, in this case, is essential for tracking individual participants, ensuring they receive the appropriate treatment, and monitoring treatment outcomes effectively. Therefore, there is no reasonable alternative that would allow the research to achieve its primary purpose of diagnosing and treating worm infections while entirely avoiding the processing of personal data.

5. Describe the steps, measures or controls you are taking to minimise the risk to privacy and safeguard the rights of the persons whose personal data you are collecting.

To minimize the risk to privacy and safeguard the rights of the individuals, particularly children, whose personal data is being collected in the research involving the diagnosis and treatment of intestinal worm infections and the development of AI-based diagnostic technology, a comprehensive set of steps, measures, and controls are taken:

1. **Informed Consent and Assent**:

    - Informed consent is obtained from parents or legal guardians for all minors (e.g. children aged 5-14).

    - Oral assent is obtained for all children, ensuring they understand the research and their willingness to participate in the research.

    - Written assent is obtained for all children aged 6 and older in Uganda, and all children aged 12 and older in Ethiopia, ensuring they understand the research and their participation.

2. **Data Minimization**:

    - Only the minimum amount of personal data necessary for the research objectives is collected.

    - Data collection is limited to essential information required for diagnosis and treatment.

3. **Pseudonymization**:

- Pseudonyms / codes are used to replace direct identifiers (e.g., names) to protect the identities of participants while allowing data tracking.

4. **Secure Data Storage and Access Controls**:

   - Personal data on paper records is securely stored by Data Protection Officers at respective sites (VCD, Uganda and Institute of Health, Jimma University, Ethiopia).

   - Personal data in electronic form is encrypted during transit and will rest in password-protected systems.

   - Strict access controls are implemented to limit data access to authorized personnel only.

5. **Data Retention Policies**:

   - Personal data will not be stored longer than necessary for research purposes. A minimum of 10 years is required in Uganda and Ethiopia as participants may be treated.

6. **Ethical Review and Approval**:

   - Ethical review and approval will be sought from relevant ethics committees or review boards to ensure compliance with ethical standards and data protection regulations. This includes Ghent University, VCD Ministry of Health Ethical Review Board and Jimma University Ethical Review Board.

7. **Data Handling Protocols**:

   - Protocols have been developed outlining the procedures for data collection, processing, storage, and disposal.

8. **International Data Transfer Safeguards**:

   - Informed Consent will be used to ensure that research participants in Uganda and Ethiopia are informed about the international transfer of their data to the EU.

   - Personal data will be collected outside of EU and brought into EU. Personal data will not be transferred outside of EU, data will be fully anonymized in these instances.

9. **Transparency**:

   - Research parties will maintain transparency with participants and their parents or guardians by clearly communicating how their data will be used. This is conveyed through the informed consent form.

10. **Secure Communication**:

    - Secure communication channels will be used for transmitting sensitive data, such as encrypted email or secure file transfer protocols.

11. **Data Encryption**:

    - All data personal data will be encrypted during transmission and storage to prevent unauthorized access.

12. **Data Anonymization or Aggregation for Reporting**:

    - Data will be anonymized and aggregated for research publication / reporting purposes / model training and evaluation to protect individual privacy while still providing valuable insights.

6. Describe the steps you have taken to make sure the research is as accurate as possible and there are minimal unintended consequences.

To ensure research accuracy and minimize unintended consequences, the follow steps have been / will be completed:

1. **Data Management Plan:** A data management plan has been created using www.dmponline.be, which outlines data handling, storage, and protection procedures.
2. **Regular Updates:** The data management plan will serve as a live document and remain up to date, reflecting any changes in data handling practices.
3. **Peer Review:** Research findings will be submitted to peer-reviewed journals for evaluation and feedback by experts in the field.
4. **Ethical Review:** Ethical approval from relevant ethics committees will ensure adherence to ethical standards.
5. **Participant Informed Consent:** Informed consent from research participants will be obtained, including guardians for minors.
6. **Random Sampling:** Random sampling techniques are employed to minimize participant selection bias.
7. **Data Validation:** Data validation checks will be implemented during data collection to maintain data quality e.g., valid age range, mandatory field checks, double entry checks, valid QR label format checks.
8. **Data Analysis:** Utilizing appropriate statistical methods and blind data analysis techniques in study protocols to reduce bias.
9. **Transparency:** Transparently documented research methodologies, data sources, and analytical approaches.
10. **Data Protection:** Personal data will be handled with strict adherence to data protection and privacy regulations.
11. **Community Engagement:** Field teams and community will be engaged to consider their perspectives and address concerns.
12. **Risk Assessment:** A risk assessment has been conducted to identify and mitigate potential unintended consequences.
13. **Reporting:** Research findings will be reported, including limitations and potential biases.
14. **Continuous Improvement:** A culture of continuous learning and adaptation will be maintained within the research team.

7. Is the (possible) negative effect or risk for the privacy of the data subjects in reasonable proportion to the processing purposes?

- Yes

8. How would you describe the likelihood of the risk(s) after having completed the previous questions in this DPIA?

- Negligible

9. How would you describe the impact of the risk(s) after having completed the previous questions in this DPIA?

- Limited

10. How would you describe the risk(s) after having completed the previous questions in this DPIA?

- Acceptable

KAKDU: EVALUATION OF AN AI-DP FOR STH DEWORMING PROGRAMS

GDPR record

GDPR RECORD

Have you registered personal data processing activities for this project?

- No

COLLECTION AND PROCESSING OF PERSONAL DATA

1. Are you collecting or processing personal data?

- Yes

2. In what format are you collecting or processing the personal data?

- Digital
- On paper

3. Are you collecting or processing primary personal data and/or secondary personal data?

- Primary personal data

4. If you are processing secondary personal data, will you inform the persons whose personal data are being processed or have they already been informed?

NA

5. If no, explain why it is impossible or why it would take a disproportionate effort to inform the persons whose personal data are being processed.

6. How will the personal data be processed?

- Pseudonymised (explain below)

The data cannot be anonymized because of the requirement to perform follow-up treatment of participants positively identified for STH/SCHm. For this reason, the key linking data to participants must be stored on paper records in a secure location for a minimum of 10 years in country.

7. If you are going to process personal data in a pseudonymised form, describe the method of pseudonymisation, where you will keep the key, and who has access to it.

Participants will be assigned a Participant ID with the following format:

- [Country Code, CC][PARTICIPANT ID, PI:0-999]
- [CC][PI]
- E.g., UG001 or ET001

This ID is used to provide pseudonymisation.
The coded Participant ID is recorded on paper records (Participant Recruitment Form), which will be stored in a secure location by authorised data controllers at respective sites:
- Vector Control Division, Ministry of health, Uganda, and
- Jimma University, Ethiopia

CATEGORIES OF PERSONAL DATA & DATA SUBJECTS

8. Are you collecting/processing any of the following special categories of data?

- Data on physical health

Participant infection status for STH/*S. mansoni*

9. Which other categories of personal data are you collecting/processing?

- Name – On paper participant registration forms only
- Age
- Sex
- Electronic location data (GPS location for school)
- Grade

10. Whose personal data are you collecting/processing?

- School aged children between the ages of 5 and 14

11. Will your research be seriously hampered if the persons whose personal data are being collected/processed exercise their right to access, to rectification, to restriction of processing, to be forgotten, to data portability and/or to object?

- No

12. If yes, please justify the need to deviate from one or more of the rights mentioned in question 11. A justification is required for each deviation.

PURPOSE(S) OF THE PROCESSING

13. What is/are the purpose(s) of the personal data processing?

1. **Clinical Assessment and Follow-Up**: Personal data, including names, schools, ages, grades, and sexes, are collected to enable the clinical assessment of participants and to facilitate necessary follow-up treatments if required. This data is essential for ensuring the health and well-being of the study participants.
2. **Training and Evaluation of AI Models**: Pseudonymized participant ID, along with age and sex information, is collected for the specific purpose of training and evaluating AI models. This data is used to develop and improve AI algorithms for diagnostic purposes, ensuring the accuracy and effectiveness of the AI microscope.

14. What is the legal ground for the processing? If the data are being processed for multiple purposes, you must describe the legal ground for each purpose.

- The research will be performed in the public interest, which means that it will lead to an increase of knowledge and insight to the direct or indirect benefit of society.
- The individuals participating in the research have freely given their explicit consent for the processing of their personal data for one or more specific purposes.

- The research is necessary for the purposes of the legitimate interests of Ghent University and/or Ghent University Hospital yet results in no high risks for the individuals participating in the research.

Additionally, the legitimate interests of Enaiblers, VCD, Jimma University.

15. If you are processing special categories of personal data (see question 8), on which exception is this based?

- The data subject has given his or her explicit consent.

GDPR RESPONSIBILITY

16. Which institution(s) is/are involved in the research?

Enaiblers

- Joint Controller
- Data Processor

Ghent University

- Joint Controller
- Data Processor

Vector Control Division, Ministry of Health, Uganda

- Joint Controller
- Data Collector

Institute of Health, Jimma University

- Joint Controller
- Data Collector

17. Is there another university, hospital, research institute or partner involved in the research (besides Ghent University and/or Ghent University Hospital)? If yes, specify below.

- Yes

18. Please specify who determines the purposes ('why') and the means ('how') of the research.

Enaiblers

- Peter Ward, peter.ward@enaiblers.com
- privacy@enaiblers.com

- Diagnostic performance evaluation and AI model development

Ghent University

- Hanne Elsen, privacy@ugent.be
- Bruno Levecke, Bruno.Levecke@enaiblers.com
- Diagnostic performance evaluation

Vector Control Division, Ministry of Health, Uganda

- Betty Nabattae, bettieful@gmail.com
- Clinical assessment and follow-up

Institute of Health, Jimma University

- Dr. Mio Ayana, Kelkiyas@gmail.com
- Clinical assessment and follow-up

DATA TRANSFERS & CATEGORIES OF RECIPIENTS

19. Are you disclosing/sharing/transferring personal data beyond your project team, either with recipients in UGent or UZ Gent, or with external recipients during or after your research?

- No

Data will be fully anonymised if disclosing/sharing/transferring to other parties.

20. If yes, to or with which categories of recipients are the personal data being disclosed/shared/transferred?

21. If yes, where are the personal data being disclosed/shared/transferred to?

22. What is/are the purpose(s) of the data transfer?

23. What is the legal ground for the data transfer? If there will be multiple data transfers, you need to indicate the legal ground for each data transfer.

RETENTION PERIOD

24. What is the envisaged retention period for the different categories of personal data? Please motivate.

Personal data must be stored by VCD and Jimma University for a minimum of 10 years due to local requirements following treatment of infected participants.

RISK ANALYSIS

25. To analyse the possible risks associated with the processing of personal data, please tick the boxes that apply to this research.

- Special categories of personal data are processed in this research (see question 8).
- The research involves innovative use or application of technological or organisational solutions, like combining the use of fingerprint and face recognition for improved physical access control.
- Personal data of children or other vulnerable persons are processed in this research (see question 10).
- Aspects concerning the data subject's performance at work, economic situation, health, personal preferences or interests, reliability or behaviour, location or movements are evaluated or scored, profiled or predicted.

26. Does the research constitute a probable high-risk processing? If you ticked two or more boxes in question 25, the answer is 'yes'.

- Yes

SECURITY MEASURES

27. What technical and organisational security measures are in place to protect personal data?

Personal Data and keys will be collected, stored and managed by Data Collectors (VCD, Jimma University). Personal data will be stored on paper records in secure locations managed by the Data Collectors.
Pseudonymised data will be used by the Data Processor (Enaiblers). Data will be collected using Electronic Data Capture application and encrypted during transfer and at rest.

28. If you have motivated the need to deviate from one or more of the rights of the persons whose personal data you are collecting/processing in question 11 and 12, please describe which safeguards are put in place to protect their rights and freedoms.
